# Restoration, conservation and phytoplankton hysteresis

**DOI:** 10.1093/conphys/coab062

**Published:** 2021-08-13

**Authors:** Maximilian Berthold, Douglas A Campbell

**Affiliations:** Department of Biology, Mount Allison University, Sackville, New Brunswick E4L 1C9, Canada

**Keywords:** phytoplankton, physiological acclimation, eutrophication, Community hysteresis

## Abstract

Phytoplankton growth depends not only upon external factors that are not strongly altered by the presence of phytoplankton, such as temperature, but also upon factors that are strongly influenced by activity of phytoplankton, including photosynthetically active radiation, and the availability of the macronutrients carbon, nitrogen, phosphorus and, for some, silicate. Since phytoplankton therefore modify, and to an extent create, their own habitats, established phytoplankton communities can show resistance and resilience to change, including managed changes in nutrient regimes. Phytoplankton blooms and community structures can be predicted from the overall biogeochemical setting and inputs, but restorations may be influenced by the physiological responses of established phytoplankton taxa to nutrient inputs, temperature, second-order changes in illumination and nutrient recycling. In this review we discuss the contributions of phytoplankton ecophysiology to biogeochemical hysteresis and possible effects on community composition in the face of management, conservation or remediation plans.

## Teaser

Humans have overloaded the world’s nutrient fluxes. Formerly clear waters are now dominated by a variety of photosynthesizing microorganisms instead of underwater plants. With insights into the diverse metabolism of these microorganisms, the chances for restoration can be improved. We summarize here the developments of recent years.

## Phytoplankton in the Anthropocene

Aquatic ecosystems, including lakes, coastal waters or marine regions, have always been subject to changes in water exchange, nutrient inflows and sedimentation. These alterations can drive replacements of species, thereby shifting community compositions and ecosystem services (Contreras-Rosales *et al.*, 2016). We now live in the Anthropocene with human activities altering terrestrial and aquatic systems worldwide (Falkowski *et al.*, 2000; Crutzen, 2002; Ellis and Trachtenberg, 2014). Aquatic systems are therefore facing new, and faster changing, combinations of environmental factors, including altered hydrological flows, elemental flows and food webs (Riegman, 1995; Cociasu *et al.*, 1996; Kemp *et al.*, 2005).

Fluxes of available carbon (C), nitrogen (N) and phosphorus (P) into the biosphere have increased by at least 13%, 100% and 400%, respectively, compared to pre-industrialized times (Vitousek *et al.*, 1997; Falkowski *et al.*, 2000; Smil, 2000). Horizontal N and P transfers are mediated in large part by industrialized agriculture, which increased N fertilization 7 times and P fertilization 3.5 times over 35 years (Tilman, 1999). These biosphere inputs will likely further increase during coming decades (Van Vuuren *et al.*, 2010), even in the face of regional countermeasures and market shifts (van Dijk *et al.*, 2016; Berthold *et al.*, 2019a). Much of this additional agricultural input will eventually wash into aquatic ecosystems, as only fractions of the fertilizer end up in the crop (e.g. Liu *et al.*, 2008). In parallel flows of sediment through rivers have been heavily regulated leading to altered inputs of silica (Si) from weathering (reviewed in Jennerjahn, 2012). Nevertheless, even in the Anthropocene seasonally and latitudinally imposed photoperiods, incident photosynthetic active radiation (PAR) and water temperatures will remain as ultimate limiting factors on future phytoplankton community compositions and distributions.

Interacting with these abiotic factors are the differential physiological capacities of species, which underlie interspecific successions over seasonal, and longer periods. Seasonal community succession patterns have been widely studied (Lampert and Sommer, 2013) and are driven by combinations of temperature (Wasmund *et al.*, 2019), current nutrient availability, water retention times (Garrido *et al.*, 2016), grazer and virus pressure (Azam *et al.*, 1983; Rohwer and Thurber, 2009). Water temperature often defines the onset and end of the growth period for a given taxon. Nutrient availability defines the carrying capacity of an ecosystem for blooms of given taxa, while grazer and virus densities interact with prey taxa densities to determine the net growth period for a taxon before it gets capped. Furthermore, (re)colonization of a taxon into an ecosystem depends upon re-growth of residual surviving cells, a local ‘seed bank’ or resting stages (Härnström *et al.*, 2011; Kremp *et al.*, 2016) or the capacity to naturally or anthropogenically (re)colonize a habitat, and perhaps form locally adapted sub-populations (Parmesan, 2006; Orsini *et al.*, 2013).

## Eutrophication

One of the direct effects of changing elemental fluxes is the human-induced eutrophication of aquatic ecosystems, which, in contrast to natural eutrophication, can occur within one human generation (e.g. de Jonge *et al.*, 2002; Gulati and van Donk, 2002; Paerl, 2006). The term eutrophication evolved over time, from an original usage for the capacity of bog systems to provide food based on their nutrient levels (Naumann, 1931). Here we define the trophic state for aquatic systems in the most general sense, as intensity of organic photoautotrophic production (*sensu*  Elster, 1958), with eutrophic systems showing a high phytoplanktonic photoautotrophic production. Eutrophication causes widespread changes not only in species composition, nutrient cycles and food web structures, but also in ecosystem services by altering commercial and recreational usage of water bodies around the globe (Lee, 1973; Vollenweider and Kerekes, 1982; Sas, 1989; Jochimsen *et al.*, 2013).

The first recognition of increased primary production was noted in alpine lakes, with algal blooms noted at the end of the 19th century, which led to losses of clear-water fish species like pike (Hasler, 1947). Available N and P, as well as their seasonal patterns, were identified as causes of this eutrophication (Gessner, 1935). Not all primary producers benefited equally from the increased nutrient concentrations. Benthic macroalgae, adapted to clear water, and low nutrients (Kufel and Kufel, 2002), were firstly replaced by macrophytes forming canopies (Verhofstad *et al.*, 2017), before most of submerged macrophytes were in turn replaced by high densities of phytoplankton (Blindow *et al.*, 1993). This shift in main primary producers, from benthos to pelagial, was described as alternative stable states, for lakes (Blindow *et al.*, 1993) and for coastal waters (Schiewer, 2007). Most recent studies suggest at least four stable states dominated by different primary producers: submerged macrophytes, emergent or floating macrophytes and then phytoplankton, each with different advantages and disadvantages regarding ecosystem services (reviewed in Janssen *et al.*, 2021).

The identification of nutrient sources and maximum allowable inputs of nutrients into aquatic ecosystems (Lee, 1973; Vollenweider and Kerekes, 1982) helped to guide restoration measures. For lakes, the Plankton Ecology Group (PEG) defined models to describe seasonal changes in lakes under increased P loading (Sommer *et al.*, 1986). The prediction of the PEG model was that depending on P status, several sequential phytoplankton blooms can appear throughout the year, with diatoms in the spring, cyanobacteria during summer and dinoflagellates in autumn. More recently the PEG model was re-evaluated and discussed against the background of global change (Sommer *et al.*, 2012), highlighting the effects of increasing temperature on overwintering and spring bloom onset, and including more food web compartments. Nonetheless, despite our increased knowledge, phytoplankton blooms, including toxic blooms, now occur more frequently and at ever shorter intervals in more and more systems (Burke *et al.*, 2001; Winder and Cloern, 2010; Winter *et al.*, 2011).

Even though factors inducing eutrophication are well understood, it is less clear how to restore eutrophied systems towards either pre-disturbance conditions or towards (re)generation of favourable ecosystem services (Xu *et al.*, 2018; Janssen *et al.*, 2021). Such favourable ecosystem services could include a large stock of fish biomass for recreational or commercial fishing, high elemental sequestration capacities (e.g. blue carbon) or non-toxic, non-nuisance species assemblages in the water column (Janssen *et al.*, 2021). Simply lowering inputs of the nutrient(s) that caused eutrophication will not necessarily lead towards some desired state (Duarte *et al.*, 2009; Jochimsen *et al.*, 2013). Such nonlinear, asymmetric responses to changing inputs, or hysteresis, derive from the internal resilience of a currently dominating community against internal and external perturbations. For example, submerged macrophyte species as dominating primary producers in oligotrophic systems sustain a set of buffering capacities against altered environmental conditions, acting as nutrient and sedimentation sinks in competition with phytoplankton (Kufel and Kufel, 2002). Likewise eutrophic systems dominated by a phytoplankton community buffer their continued dominance by, for example, lowering light availability for benthic primary producers (Scheffer *et al.*, 1997) or inorganic carbon availability for competing phytoplankters (Dam *et al.*, 2018).

Therefore, to inform conservation and management goals, we review interactions of phytoplankton ecophysiology with our world of global change, through literature spanning both field and laboratory experiments. We hope to support management decisions in the face of multiple ecosystem stable states (see Table 1).

**Figure 1:**
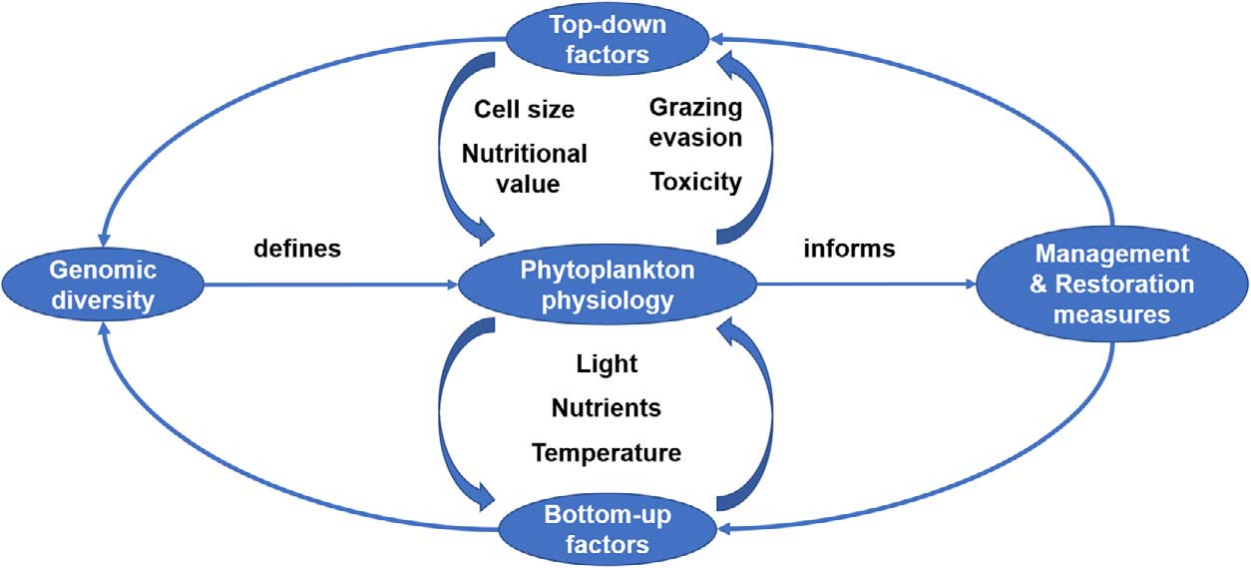
Importance of phytoplankton physiology defined by genotype and phenotype in restoration and management activities.

## Biogeochemical and Trophic Hysteresis

### Bottom-up and top-down effects of phytoplankton blooms

Phytoplankton blooms can affect the ecosystem bottom-up by decreasing euphotic depths, and thereby lower O_2_-production at depth while simultaneously boosting O_2_-consumption below the euphotic zone. The spread of hypoxia in freshwater systems is directly linked to human influences, through for example increased nutrient supply (Jenny *et al.*, 2016). This elevated net O_2_-demand at depth can lead to the loss of parts of the macrozoobenthos (Riemann *et al.*, 2016), and affects the nutrient sequestration capacities of sediments. Suboxic sediments lose their capacities for storing P and Fe (Lampert and Sommer, 2007; Molot *et al.*, 2014), which depend upon redox poise. Simultaneously suboxic sediments increase their denitrification capacities (Gruber and Sarmiento, 1997; Hietanen and Lukkari, 2007; Deutsch *et al*., 2010) as competition from aerobic respiration is suppressed. The resulting shift towards lower available N:P ratios and possibly higher Fe availability stimulates N-fixing species like cyanobacteria and helps keep the system within a cycle of internal fertilization (Lampert and Sommer, 2007; Molot *et al.*, 2014) through water-column N fixation and P and Fe (re-)releases from sediments. This biogeochemical legacy effect can prolong system recovery by several decades after nutrient inflows are lowered to pre-disturbance concentrations (Chorus *et al.*, 2020). Contrary, if eutrophic systems stay oxygen saturated, phytoplankton may experience Fe limitation (Geiß *et al.*, 2004), especially in brackish waters with high co-precipitation capacities for Fe and P (Gunnars *et al.*, 2002; Gunduz *et al.*, 2011). If nutrient inflows are not lowered sharply, the post-eutrophication community may persist indefinitely (Nürnberg, 2009; Chorus and Spijkerman, 2021), in a hysteresis generated by biogeochemical interaction. Systems with such incomplete management actions remain in an unfavourable state, and knowledge on ecophysiological mechanisms may inform stakeholders about further steps (Fig. 1).

**Table 1:** Summary of phytoplankton hysteresis and potential interventions

External driver	Consequence	Solution	References
Increasing DOC	Increasing re-mineralization in eutrophic waters	Decrease erosion and other nutrient inflows	Weyhenmeyer *et al.*, 2014; Nieminen *et al.*, 2018; Li *et al.*, 2019b
Low NO_3_^−^:NH_4_^+^	Toxic HAB	Decrease of NH_4_^+^ input and maintenance of NO_3_^−^ to decrease sediment P resolubility	Wauer *et al.*, 2005; Newell *et al.*, 2019
Low DIP:DOP ratio	High APA turnover and source shift	Decrease overall P inflow and release from sediment	Zhang *et al.*, 2017; Chorus *et al.*, 2020
Low Si inflow	Vanishing of diatom blooms	Increase fluvial matter transport	Cociasu *et al.*, 1996; Jennerjahn, 2012
High frequency of pulse disturbance	Fast-exploiting resilient blooms	Change pulse to press disturbance	Klappenbach *et al.*, 2000; Shade *et al.*, 2012
Bloom persistence after nutrient input drop	Pulse-mediated stable phytoplankton	Determine and lower non-point sources and organic nutrient pools	Berthold *et al.*, 2018; Li *et al.*, 2019a; Chorus *et al.*, 2020

Phytoplankton blooms also affect the top-down control of ecosystems. Food webs are affected when phytoplankton secondarily provoke the loss of submerged vegetation through shading (Blindow *et al.*, 1993; Scheffer *et al.*, 1997). At the level of phytoplankton community composition blooms can provoke a drop, or variable abundances, of so-called high-quality phytoplankton that contain high amounts of polyunsaturated fatty acids (Taipale *et al.*, 2019). There are also grazer-mediated changes in cell size and grazer-selection of non-toxic strains (see below, Ger *et al.*, 2019; Lürling, 2020). Less palatable phytoplankton strains can then persist in a new stable state with lower grazing, in a hysteresis generated by trophic interactions.

### Post-eutrophication restoration

Phytoplankton blooms and community structures can therefore not always be predicted from simple nutrient input–output models but are rather influenced by the physiological responses of diverse phytoplankton taxa to altered nutrient supply, temperature, second-order changes in illumination (Sas, 1989) and possibly to dissolved inorganic carbon (DIC). Therefore, interactions of biogeochemical and trophic mechanisms need to be considered when restoring phytoplankton-dominated systems (Ibelings *et al.*, 2007). Depending upon the desired conservation or management goals, results of restoration may differ from the pre-disturbance ‘reference condition’, ranging from minimally disturbed to best attainable conditions (Stoddard *et al.*, 2006) or possibly a desirable state distinct from the pre-disturbance reference condition. Current management strategies aim to lower nutrient fluxes into eutrophied systems (Paerl *et al.*, 2011), with the focus on P, or N and P simultaneously, while bearing in mind economic feasibility (Conley *et al.*, 2009; Lewis *et al.*, 2011; Molinos-Senante *et al.*, 2011).

Ultimately, P is assumed to be the limiting nutrient for most limnic systems (Liang *et al.*, 2020), while an N and P colimitation is common for coastal water bodies (Howarth and Marino, 2006). However, as N can be fixed from the atmosphere, management controls on water-borne P may be more feasible even in coastal systems, often in conjunction with upstream management of P for limnic systems. Nonetheless, aeolian and rain-borne P inputs can be substantial depending on regional land use (Tipping *et al.*, 2014; Berthold *et al.*, 2019b). In different habitat types including lakes and coastal water bodies, managed nutrient declines have not always led directly to improvements in water quality, species composition or system stability (Schindler, 2006; Conley *et al.*, 2009; Schindler *et al.*, 2016). Such delayed ecosystem reactions depend partly on the managed drops in external nutrient loading being countered by re-release of sequestered nutrients, particularly P, and can last decades (Nürnberg, 2009; Schindler *et al.*, 2016; Chorus *et al.*, 2020).

Shallow lakes, coastal ecosystems and even ocean basins (North Sea, Baltic Sea) may thus experience prolonged disruption during post-eutrophication, with increased Chl a, despite managed drops of total N (TN) and total P (TP) within the water column (Gulati and van Donk, 2002; McQuatters-Gollop *et al.*, 2007; Schiewer, 2007). For example, in Lake Constance observed shifts in total phytoplankton biomass and composition happened rapidly only after a certain nutrient threshold was reached, with a simultaneous change from dominance by cyanobacteria to chrysophyte dominance (Jochimsen *et al.*, 2013). Over a longer time frame of decades, the central basin of the Baltic Sea showed a shift in phytoplankton spring bloom composition from diatoms to dinoflagellates after lowering of external nutrient inputs (Hjerne *et al.*, 2019). Coastal water bodies are challenging to manage as they usually receive the complete load of a catchment area and are especially prone to eutrophication-related problems. For such systems, biomanipulation has been proposed to alter the food web structure to increase grazing pressure on phytoplankton (Fonseca *et al.*, 1994; Schiewer, 2007), as for example ongoing re-planting of submerged vegetation (van Keulen *et al.*, 2003) or removal of planktivorous and stocking of piscivorous fish (Jeppesen *et al.*, 2007). Biomanipulation through changes in fish composition are discussed, but often fail if the overall nutrient inputs are not reduced accordingly (Søndergaard *et al.*, 2007). Fish stock manipulations need to be constantly re-applied (~10 years) and may alter energy flow and fish species composition in the long term (Syväranta *et al.*, 2011; Rogers and Allen, 2012). These methods depend therefore as much upon parallel restoration measures within the catchment as upon the application of the biomanipulation. Other post-eutrophied systems developed communities of grazing resistant species upon mineral nutrient declines that lowered food web efficiency (Schiewer, 2007). Selective grazing by *Daphnia* can promote dominance of remaining toxic *Microcystis* (Ger *et al.*, 2019), and furthermore different genotypes of *Microcystis* show different susceptibilities to grazing (White *et al.*, 2011). This example emphasizes the importance of future studies on the impacts of zooplankton diversity on phytoplankton diversity. Thus, restoration measures can take anything from years to decades to be effective and may generate undesired outcomes.

## Hysteresis through Ecophysiological Acclimation

The biogeochemical and trophic responses of phytoplankton communities involve members of at least 12 eukaryotic phytoplankton lineages and the ancient, diverse lineage of prokaryote cyanobacteria. Each of these lineages in turn comprises numerous genotypes, encoding numerous combinations of metabolic and physiological responses. It is thus difficult to define general practices to treat ‘nuisances’ like harmful algal blooms or undesired stable community states. When facing changing environmental conditions, individual cells rely on differential expression of their genotype to alter their phenotype (Fig. 1). When individual genotypic capacities for phenotypic acclimation are exhausted, other individuals with a slightly different genotype from the same population can be favoured or communities may shift composition towards taxa with different genotypes. After the overall community capacity to acclimate is exhausted, when conditions no longer support net growth, new sets of taxa may progressively dominate in a move towards a different community state, which will in its turn display acclimatory resilience in the face of external pressures. Of course, pressure on a specific phenotype is always a pressure on the genotype, which can cause evolutionary adaptation to the new environmental state on a generational timescale, within genomic or cytological limits for given taxa (Thomas *et al.*, 2012; Reusch and Boyd, 2013; Aranguren-Gassis *et al.*, 2019).

Physiological acclimations include changes in buoyancy, nutrient uptake rates, orientation and content of light-harvesting pigments, cellular composition, enzymatic activities and use of different nutrient and energy sources, among other responses to temperature, light and nutrients. Such acclimations can happen over times of minutes to hours and are highly reversible, depending on the nature, amplitude and duration of the environmental signal.

### Temperature acclimation

Temperature interacts on multiple levels with cell metabolism. Temperature, per se, is largely beyond management influence, but interacts strongly with other factors that are susceptible to management. Phytoplankton community growth rates show a response to a 10°C temperature increase (*Q*10) of 1.4–1.9 (Eppley, 1972; Bissinger *et al.*, 2008; Edwards *et al.*, 2016; Sherman *et al.*, 2016), but only if other resources are not growth limiting. Increasing water temperatures will therefore be of limited direct importance in enhancing community growth rates in subtropical to temperate regions if nutrients in oligotrophic regions, or light in turbid or eutrophic regions, remain as limiting factors (Edwards *et al.*, 2016; Marañón *et al.*, 2018). Temperature increase can, however, affect the phytoplankton community species composition of temperate waters within days, by favouring, e.g. green algae or cyanobacteria (Weisse *et al.*, 2016). With increasing temperatures an increase of blooms in shallow waters can be expected (Trombetta *et al.*, 2019), as well as an earlier onset of blooms during spring and later autumn blooms (Wasmund *et al.*, 2019). Several phytoplankton genotypes isolated from the tropics are already growing close to their maximum temperature, with risk of diversity losses with further increases in temperatures (Boyd *et al.*, 2013). In polar regions water temperature strongly shapes the current phytoplankton composition by limiting enzyme turnover rates (Young *et al.*, 2015) and membrane function (Nishida and Murata, 1996). Also, in polar regions, current species may be psychrophilic strains adapted to only cold temperatures or psychrotrophic strains that tolerate low temperatures. Diatoms can dominate cold waters, and most of these polar diatom species are psychrophilic strains (Suzuki and Takahashi, 1995; Boyd *et al.*, 2013; Lacour *et al.*, 2017). Even small increases in water temperature could profoundly influence future community compositions in such colder waters.

Acclimation and adaptation in diatoms rely on investments in key physiological mechanisms that determine growth temperature optima among ecotypes (Liang *et al.*, 2019). Cold waters usually transport oxidized forms of N, like NO_3_^−^, as a result of upwelling events. Diatoms and dinoflagellates in particular prefer NO_3_^−^ (reviewed in Glibert *et al.*, 2016), possibly because the enzyme nitrate reductase shows highest efficiencies at 5–25°C (Fan *et al.*, 2003). These cold upwelling regions are thus niches for large, sinking-sensitive species (Cloern, 1996, 2018), as other phytoplankton groups grow better at higher temperatures and preferentially use different N sources. Interestingly, cyanobacteria seem to be absent from most cold-water marine areas (Vincent, 2000), yet dominate as mats or picoplankton in cold oligotrophic freshwater systems (Vézina and Vincent, 1997). Furthermore, these freshwater cyanobacteria seemed to be cold temperature tolerant rather than adapted (Vincent, 2000). These differences in strategies will influence the shape of future polar phytoplankton assemblages, as strains with wider temperature tolerance start to dominate with increasing water temperature at least in summer, whereas psychrophilic strains will be replaced, displaced poleward or seasonally restricted, leading to diversity shifts around the globe (Thomas *et al.*, 2012; Barton *et al.*, 2016). The acclimatory capacity of a phytoplankton assemblage, or meta-populations of several genotypes, depends on the intraspecific diversity. Recent studies with Arctic isolates suggest that a large intraspecific diversity may be as important as interspecific diversity for adaptation and selection in phytoplankton assemblages (Wolf *et al.*, 2018). Diversity loss might lower the resistance of current phytoplankton communities to change while favouring species able to exploit decreasing nutrient levels (Allison and Martiny, 2009).

### Photoacclimation

Photoacclimation comprises a complex, widely studied network of responses to changing light availability (Falkowski and Owens, 1980; Falkowski *et al.*, 1981; Behrenfeld *et al.*, 2002; Graff *et al.*, 2016). From the view of wider conservation and ecosystem services photosynthesis leads to fixation of C and release of O_2_, which in turn affect all trophic levels and nutrient cycles. Across taxa, cells have variable capacities to control their internal pigment content (Graff *et al.*, 2016) and even their pigment orientation to incoming light (Schuergers *et al.*, 2016) to either protect themselves from oversaturating conditions (Bailey and Grossman, 2008) or to counter lower light availability (Geider, 1987). These photoacclimatory strategies interact strongly not only with availability of the nutrients needed to assemble the photosynthetic system (see below), but also with cell size. Small phytoplankton cells show a smaller self-shading (or packaging) effect at the cellular level, as there is not enough space (Ting *et al.*, 2002) to stack large amounts of pigments within a small cell (Finkel, 2001). The lower packaging effect in small cells leads to a higher absorption cross section per pigment or per reaction centre, and therefore higher light absorption per pigment or per nutrient investment (Kirk, 1975). A community of small cells can thus pre-empt larger cells through better photosynthetic return upon limited nutrient investment, with *Prochlorococcus* in the open ocean a case in point. On the other hand, such small cells are intrinsically more prone to photoinactivation because of their same limited optical thickness (Key *et al.*, 2010; Campbell and Serôdio, 2020) and can thus suffer under variable light conditions.

The amplitude and rapidity of short-term acclimation to changing light varies widely across taxa, conferring differential capacities to withstand and exploit low or fluctuating light (Raven, 2011), thereby imposing different limits on the conditions where photosynthesis remains a viable strategy for different taxa (Murphy *et al.*, 2017). Accumulation of cells in a community increases light attenuation, and the relation between biomass and light attenuation varies across taxa (Scheffer *et al.*, 1997; Kemp and Villareal, 2018). Therefore, phytoplankton growth can itself impose light limitation on competing taxa, along with steep light attenuation gradients that secondarily generate a variable light environment. The growth of taxa able to exploit such low or fluctuating light can then maintain an alternate stable state by preventing the (re-)occurrence of other phytoplankton taxa or submerged macrophytes, which need higher or more stable light.

The accumulation and maintenance of the photosynthetic system also imposes differential material (Finkel *et al.*, 2010a) and energy costs across phytoplankton taxa. Some phytoplankters adopt a ‘just in time’ strategy of (re)building photosynthetic complexes rapidly, as required, which requires higher investment of energy into the metabolic systems to turn over proteins (Lavaud *et al.*, 2016; Bonisteel *et al.*, 2018). In contrast other phytoplankton adopt a ‘warehouse’ strategy of investing in excess stocks of the key proteins to support re-assembly of photosynthetic complexes (Campbell *et al.*, 2013; Ni *et al.*, 2017), which imposes a higher standing elemental investment, lower metabolic return upon invested nitrogen, but less immediate demands upon energy metabolism. These costs of accumulating and maintaining the photosynthetic system also interact with available nutrient levels (Loebl *et al.*, 2010; Li *et al.*, 2015) (see ‘Nitrogen’ below) thereby creating differential selections upon different taxa. Temperature strongly limits performance of the photosynthetic complexes and phytoplankton must increase their resource allocations to key metabolic complexes to maintain key fluxes at low temperature (Young *et al.*, 2015; Ni *et al.*, 2017).

At a higher level of organization, upon achieving dominance, filamentous cyanobacteria disproportionately lower available light in lakes (Scheffer *et al.*, 1997), through for example the high packaging effect of colonies that cause shading (Tilzer, 1987). This high shading within colonies can then support reducing micro-environments, which are more favourable for N fixation (Tilzer, 1987). Furthermore, the underwater light climate in shallow waters can be lowered to a point that the largest part of the water column remains net-heterotrophic, which favours species that can acclimatize fast to changing light (Berthold and Paar, 2021). In summary, differential capacities for photoacclimation contribute to niche partitioning across phytoplankton (Six *et al.*, 2007a). It is intriguing that the prokaryotic cyanobacteria present by far the widest spectral diversity among phytoplankton lineages with distinct spectral profiles even within closely related taxa (Six *et al.*, 2007b), in comparison to brown or green colours prevailing across most other lineages. Somewhat unexpected correlations among particular pigment gene clusters and adaptation to brackish environments show that spectral competition may be a major factor establishing niche boundaries (Larsson *et al.*, 2014; Grébert *et al.*, 2018).

### Nutrient acclimation

Acclimation to changing nutrient concentrations is crucial to sustain growth rates and to buffer short-term limitations. Acclimation processes are not necessarily taxa specific, but rather depend on phenotypic plasticity, including changes in cell size, changes in cellular reserves or genetically encoded high- and low-affinity nutrient uptake systems. Nutrient limitation in field vs. laboratory studies is a highly controversial topic, since in principle minimum uptake thresholds for nutrients must be defined for certain taxa.

The regulation of uptake rates depends on the type of nutrient and storable reserves within cells, i.e. the cell quota *Q* (Droop, 1973). Cell sensing for limiting nutrients is regulated through the availability of the nutrient within the cytoplasm. Upon experience of altered nutrient amounts within the cell, phytoplankton cells react with short-term and long-term transcriptional changes. There are both high- and low-affinity transporter classes for N, P and Si with expression and therefore uptake rates depending on internal nutrient concentrations or elemental ratios (Thamatrakoln and Hildebrand, 2008; Glibert *et al.*, 2016; Lin *et al.*, 2016).

### Nutrient ratios and competition

Beyond absolute availability thresholds for individual nutrients, elemental ratios that imply nutrient limitation have been derived from an empirical elemental ratio of C:N:P for particulate matter in the ocean (Redfield *et al.*, 1963). This Redfield ratio concept has been widely used to describe apparent nutrient limitations of phytoplankton biomass (Elser *et al.*, 1990; Hessen *et al.*, 2013; Lee *et al.*, 2015). Field ecologists usually apply the ratio of total nitrogen to total phosphorus (TN:TP) or the ratio of dissolved fractions (DIN:DIP) considering only directly measurable inorganic fractions, such as nitrate, ammonium or ortho-phosphate, to infer whether N or P is a limiting nutrient for phytoplankton growth. However, nutrient ratios are taxon specific and may even depend on the global availability of nutrients (Geider and La Roche, 2002; Finkel *et al.*, 2010b; Talmy *et al.*, 2014). For example, due to higher availability of iron and its possible stimulation of nitrogen fixation during glaciation, the Redfield ratio may once have been 25 N:1 P instead of the current 16:1, (Broecker and Henderson, 1998). Nutrient limitation predictions based on the empirical Redfield ratio may therefore only represent the current most widely distributed taxa and their average elemental composition, which can change in the future, across habitats or with shifts in community composition.

In laboratory experiments nutrient requirements follow ratios that are species and size specific (Marañón *et al.*, 2013). For example, depending on cyanobacterial taxon N:P ratios can range from 13:1 to 50:1 (Finkel *et al.*, 2010b). However, it is not clear if high N:P ratios in cyanobacteria derive from adaptation of at least some strains to generally lower P availabilities in oligotrophic ocean regions, therefore skewing the taxonomic interpretation of the ratio. Picocyanobacteria from oligotrophic parts of the ocean replace P with S in their cell membrane lipids (Van Mooy *et al.*, 2006) to lower their P demand. Furthermore, N-fixing cyanobacterial species start to dominate at low N:P ratios (Karl *et al.*, 2002). However, the occurrence of N-fixing cyanobacteria is not necessarily linked to low N:P ratios, as they can also occur at high N:P ratios, limiting generalizations (Chislock *et al.*, 2014). Therefore, if certain N:P ratios could lead to exclusive domination by one or another group, that fails to account for the diversity of phytoplankton in aquatic ecosystems. This diversity is in part explained by a resource-based competition theory, where different species can coexist, if they are limited by different resources (Tilman, 1977), explaining diversity through chaotic conditions by resource competition for more than three resources (Huisman and Weissing, 1999). This interpretation may be complicated if cells limited by one resource engage in luxury consumption of another resource limiting to other cells. Larger cyanobacteria, like *Nostocales, Aphanizomenon* or *Nodularia*, can, for example, luxury consume P and store it, rather than use it directly for growth (Vahtera *et al.*, 2010; Hagemann *et al.*, 2019). Similarly, diatoms like *Chaetoceros* and *Ceratium* can luxury consume NH_4_^+^ at high rates, even at low DIN concentrations (Olofsson *et al.*, 2019). This ‘ravenous’ nutrient uptake may prevent the seasonal or annual occurrence of other phytoplankton taxa (de Mazancourt and Schwartz, 2012).

Long-term drops in nutrients can favour a community shift towards dominance by mixotrophs such as chrysophytes (Jochimsen *et al.*, 2013), which can alleviate nutrient limitation through mixotrophy (Lewitus and Kana, 1995; Krom *et al.*, 2005). Bacterioplankton competes directly with phytoplankton for N and P (Joint *et al.*, 2002), but bacterioplankton can be subsequently grazed by mixotrophic phytoplankton, especially under limited light and increased temperature (Wilken *et al.*, 2018). Monitoring phytoplankton diversity is therefore necessary not only to identify harmful taxa, but also to track highly competitive bloom-stabilizing taxa and shifting life strategies.

#### Carbon

DIC is rising as a result of anthropogenic releases of CO_2_ (Royal Society (Great Britain), 2005). Phytoplankton responses to this increase in DIC are complex and diverse, varying with taxa and interacting with other environmental factors (Gao *et al.*, 2012; Gao and Campbell, 2014), placing predictions of potential winning and losing taxa under higher pCO_2_ beyond the scope of this review. A perhaps unexpected secondary effect of eutrophication is, however, the onset of intermittent limitations upon phytoplankton growth by lowered DIC in some habitats, including estuaries (Fogel *et al.*, 1992) as well as in brackish waters during red tide episodes (Hansen *et al.*, 2007) or hypereutrophic lakes (Dam *et al.*, 2018). Rapid phytoplankton growth can, under such conditions, place local limitations upon the delivery of DIC into cells for assimilation.

DIC limitation directly limits the assimilation of carbon and thus imposes feedback limitations upon photosynthetic electron transport. DIC limitation also interacts with requirements for other nutrients, altering cellular responses to changing nitrogen availability (Barker-Aström *et al.*, 2005). Although many freshwater taxa have multiple systems for uptake and accumulation of DIC (Raven, 2003; Raven *et al.*, 2014), some marine taxa, which evolved for life under stable, high DIC and pH, have only limited systems for DIC accumulation encoded within their genomes (Price *et al.*, 2008; Shen *et al.*, 2017). Thus, eutrophication of a habitat can, secondarily, alter the prevalent DIC regime from a high, stable geochemically defined DIC to a lower, more variable DIC under dynamic biogenic influence, thereby imposing at least intermittent limitations upon growth of some phytoplankton taxa. These effects may interact with changing delivery of dissolved organic carbon (DOC), as increasing numbers of storms may transport pulses of excess DOC into water bodies (Asmala *et al.*, 2021). Such elevated DOC can persist up to 200 days and can be remineralized faster through bacterial respiration under elevated P inputs (Allesson *et al.*, 2020). Nutrient-rich water bodies may therefore act as net-carbon sources, as more C-compounds can be remineralized rather than sequestrated in the sediment (Thingstad *et al.*, 2008). Monitoring of the biological oxygen demand after addition of N, or P, might be a feasible approach for state agencies to identify such seasonal C-source conditions within an ecosystem (Mallin *et al.*, 2004; McCormick *et al.*, 2006).

In general, coastal and bloom-forming taxa may be pre-adapted to cope with moderate decreases in DIC and pH, through their evolution under the influence of short-term biogenic fluctuations (Li *et al.*, 2016). Biogenic shifts in carbonate regime can thus act to exclude taxa adapted to high, stable DIC from persisting in environments with fluctuating DIC and thereby contribute to a hysteresis of phytoplankton community responses to changing nutrient inputs (O’Neil *et al.*, 2012; Dam *et al.*, 2018).

#### Nitrogen

There are NH_4_^+^ and NO_3_^−^ transporters, with either high or low affinities (reviewed in Glibert *et al.*, 2016). The availability of NH_4_^+^ lowers the activity of NO_3_^−^ reducing enzymes, but NO_3_^−^ does not, however, lower NH_4_^+^ assimilation, because NO_3_^−^ necessarily passes through NH_4_^+^ during assimilation (Dortch, 1990). Furthermore, NH_4_^+^ is sometimes considered a preferred N source for phytoplankton, but concentrations above 30 μmol l^−1^ can be growth suppressing (Glibert *et al.*, 2014). NH_4_^+^ uptake is therefore regulated with fewer NH_4_^+^ transporters at increasing NH_4_^+^ concentrations (Post *et al.*, 2012). The ratio of available NO_3_^−^ to NH_4_^+^ can further affect phytoplankton community composition, as NO_3_^−^ assimilation enzymes are cold-temperature adapted (see above; Fan *et al.*, 2003). Contrarily, higher NH_4_^+^ and temperature can lead to dominance by cryptophytes and cyanobacteria (Glibert *et al.*, 2016). This selective pressure was observed to act seasonally on phytoplankton compositions in eutrophic reservoirs (Andersen *et al.*, 2020) and even over decades in lake fertilization experiments (Swarbrick *et al.*, 2019). Low NO_3_^−^:NH_4_^+^ can therefore promote harmful cyanobacterial blooms (Newell *et al.*, 2019). On the other side, in systems with high but fluctuating NO_3_^−^:NH_4_^+^, larger taxa with vacuoles tend to dominate, as stored NO_3_^−^ does not inhibit uptake rates (Stolte and Riegman, 1995).

**Figure 2:**
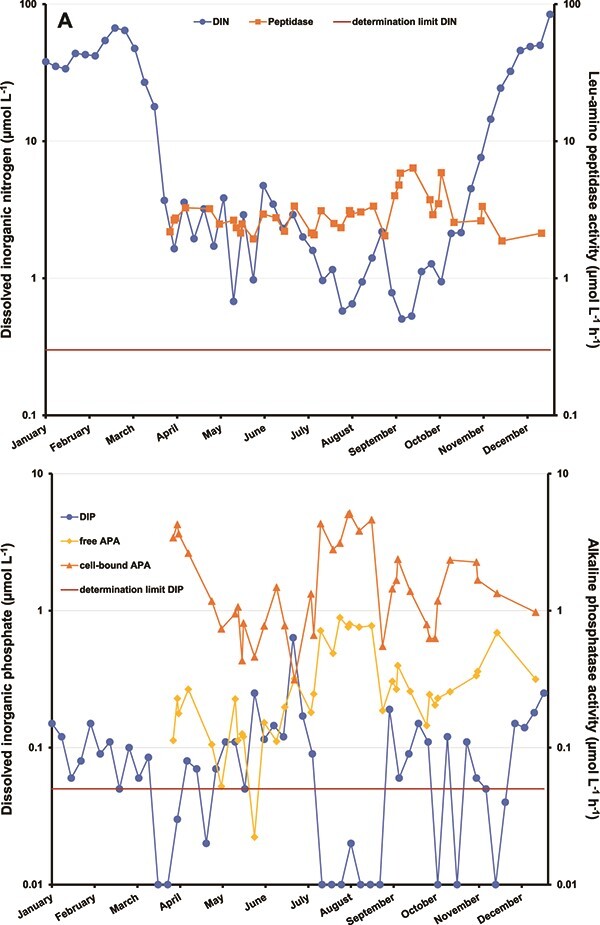
Example of seasonal activity of either extra-cellular or membrane-bound enzymes in the eutrophic Darss-Zingst lagoon system. Enzymatic activities are plotted along with the weekly average amount of dissolved inorganic nitrogen (A, sum of ammonium, nitrate, nitrite) and DIP (B, includes fraction of soluble reactive phosphorus).

Picocyanobacteria use different (N-free) compatible solutes for salinity acclimation, as an adaptation to different N limitation (Klähn *et al.*, 2010). Prolonged N limitations can furthermore induce the production of N-scavenging enzymes (see Fig. 2A), including amino acid oxidases and proteases, either membrane bound or extracellular (see list in Berges and Mulholland, 2008) to access dissolved organic N (DON).

DON is an important N source for phytoplankton but is difficult to characterize as it consists of complex, partly undescribed molecules within the pool of dissolved organic matter (Mulholland and Lomas, 2008). Autochthonous DON derived from phytoplankton was found to be the preferred DON source for natural phytoplankton assemblages, whereas bacterioplankton preferred allochthonous DON material (Korth *et al.*, 2012). Recycling of DON prolongs support of phytoplankton growth before N forms eventually exit the system through pathways of denitrification or anaerobic ammonium oxidation (Deutsch *et al.*, 2010; Jäntti *et al.*, 2011).

N-fixing cyanobacteria can overcome the onset of N-limitation by developing heterocyst or by scheduling N fixation during the nighttime (Toepel *et al.*, 2008; Grover *et al.*, 2020) to limit inhibition of nitrogenase by the O_2_ released from photosynthesis. An increase in the *HetR*-protein is responsible for heterocyst development within 3 hours of the onset of N limitation, with the N status intracellularly sensed by changing levels of 2-oxoglutarate (Zhou *et al.*, 1998; Muro-Pastor *et al.*, 2001). In species with heterocyst, the amount of heterocyst per colony is therefore a good estimator of bloom reliance on N_2_, as cyanobacteria can dominate by acquiring other N sources (Ferber *et al.*, 2004; McCarthy *et al.*, 2013). The release of excess fixed N from N-fixing cyanobacterial blooms further stimulates other phytoplankton compartments and the whole food web (Karlson *et al.*, 2015), further increasing the ecosystem N burden even after an initial cyanobacterial bloom collapse.

N limitation interacts with upkeep of photosynthesis since photosynthetic pigments and catalytic complexes represent major standing investments of N (Li *et al.*, 2015), which can accumulate well above instantaneous requirements for catalysis of photosynthesis. These large pools can then support generations of cell division without net accumulation of new protein, enabling cells to temporally offset biosynthetic accumulation of key proteins from rounds of subsequent division after dissolved N resources are depleted. Cells thereby indulge in luxury accumulation and sequestration of N resources to support later generations of growth, excluding other taxa from exploiting the habitat (de Mazancourt and Schwartz, 2012). In turn, the N costs and investments to maintain photosynthesis in a taxon shifts depending upon other external factors, notably including pCO_2_ (Li *et al.*, 2015). Other strategies to cope with N limitation, for example loss of pigments (chlorosis), support survival of intermittent limitations under unfavourable conditions (Barker-Aström *et al.*, 2005; Klotz *et al.*, 2016).

Management efforts should therefore focus on lowering NH_4_^+^ (Newell *et al.*, 2019), possibly in conjunction with maintenance of NO_3_^−^ in deeper, stratified waters to pre-empt redox-sensitive P reflux from sediments (Wauer *et al.*, 2005). Simultaneous lowering of P should be considered, as most toxic N-fixing species thrive at elevated P concentrations during high temperatures and at low NH_4_:NO_3_-ratios (see above and following). Inorganic N will eventually be removed from the system through various microbial pathways, but DON may remain accessible to phytoplankton. Thus, organic N sources should be preferably lowered instead of focusing solely on total N (Lewis *et al.*, 2011).

#### Phosphorus

The thresholds for P-deficiency responses range from 0.1 to 0.4 μmol ortho-P L^−1^ (dissolved inorganic phosphorus, DIP) in laboratory cultures, lakes, lagoons and marine waters (Grillo and Gibson, 1979; Nausch, 1998; Li *et al.*, 2019a; Berthold and Schumann, 2020). This threshold range for P deficiency is consistent with growth saturating concentrations ranging from 0.4 to 0.8 μmol l^−1^ measured in laboratory cultures (Grover, 1989). However, these apparently high concentrations for onset of P deficiency are in stark contrast with low, sometimes undetectable P concentrations in lakes, coastal waters and even oceans during summer or in general (Hudson *et al.*, 2000; Martin *et al.*, 2014; Berthold *et al.*, 2019a).

Environmental samples contain, however, a variety of dissolved organic P (DOP), which is only partly accessible through the colorimetric determination used to determine soluble reactive phosphorus (SRP). DOP concentrations are rarely monitored, but they can make up to 99% of dissolved P fractions (Karl and Björkman, 2015). This fraction is assumed to support phytoplankton growth even under non-detectable DIP or SRP concentrations.

Ultralow concentrations of P in lakes imply high turn-over rates of P among cells (Hudson *et al.*, 2000). However, P uptake is an energy-demanding process and very low P concentrations can be at the threshold of thermodynamically feasible P uptake (Grillo and Gibson, 1979; Ritchie *et al.*, 2001). Uptake of PO_4_ in cultures of *Synechococcus* R-2 is ATP driven with a demand of one ATP per molecule PO_4_ (Ritchie *et al.*, 2001). It is assumed that the very low traces of P found in oligotrophic lake water or growth media represent a steady state between cells and surrounding media, due to P leakage or turn-over (Hudson *et al.*, 2000; Isvánovics *et al.*, 2000).

To overcome persistent nutrient limitation, cells can produce cell-bound and extracellular phosphatases, as a second-tier response, up-regulated as the cell P quota drops below the level at which high-affinity transporters are expressed (Litchman and Nguyen, 2008; Ostrowski *et al.*, 2010). It is possible that under severe P-limiting conditions, P uptake happens so fast that every P molecule released from DOP by alkaline phosphatase activity (APA) is taken up nearly instantaneously, leaving no detectable DIP in the water column (Wynne and Rhee, 1988; Hudson *et al.*, 2000; Berthold and Schumann, 2020; Fig. 2).

P turn-over mediated by phosphatase can be quite high and is usually negatively correlated with available dissolved inorganic P (Nausch, 1998). Such high activities can turn over the complete pool of DOP in 11 hours to 200 days, depending on biomass, season and available PO_4_^3−^ (Nausch, 1998; Labry *et al.*, 2005). Total phosphatase activity depends additionally on the amount of bacterioplankton in the water column. Indeed, it is not technically possible to discriminate between the phosphatase activities from phytoplankton and bacterioplankton using the common bulk community assay of 4-methylumbelliferyl phosphate-fluorescence and even size-class filtration prior to the assay may show some carry-over effects (Hoppe, 2003; Labry *et al.*, 2005). A possible approach is enzyme-labelled fluorescence markers and the use of an epifluorescence microscope (González-Gil *et al.*, 1998; Dyhrman and Palenik, 1999; Štrojsová *et al.*, 2003). However, this approach is time-consuming. Furthermore, long-term data sets on DOP are rare (van Beusekom and de Jonge, 2012; Karl and Björkman, 2015), as DOP is not usually monitored as part of water quality programs. Including DOP would be a feasible variable for monitoring agencies to better determine actual P supply status within a system, compared to e.g. enzyme kinetics assays or epifluorescence microscopy.

In terms of anthropogenic control phosphatase production and stability is light dependent, in that light is necessary for phytoplankton to produce phosphatase (Wynne and Rhee, 1988). At the same time, however, phosphatase stability decreases upon exposure to light and shading by DOM can protect phosphatase from degradation (Janssen and McNeill, 2015), allowing more prolonged phosphatase activity. Such DOM is either allochthonous or autochthonous. Scenarios in lakes point towards an increased brownification through increased DOM and iron (Weyhenmeyer *et al.*, 2014), leading to possibly enhanced phosphatase half-life times, and therefore increased capacity for P recycling. This enhanced P recycling in the water column could then in turn favour bacterioplankton and phytoplankton communities and sustain their biomass, possibly prolonging their post-eutrophication persistence.

Cells can form PolyP granules even against strong concentration gradients, as described for lakes, oceans and cultures (Martin *et al.*, 2014; Diaz *et al.*, 2019; Hagemann *et al.*, 2019; Li *et al.*, 2019a). Under P-limiting conditions phytoplankton assemblages may show a luxury consumption of P as a response to previous deficiency. There seems to be no size pattern, as filamentous cyanobacteria, as well as picoplankton can accumulate large amounts of PolyP (Hagemann *et al.*, 2019; Li *et al.*, 2019a). Picoplankton recycling of PolyP seems to support other blooming algae in eutrophic waters of Lake Ontario, therefore mediating P fluxes from smaller to larger phytoplankton (Li *et al.*, 2019a). Uptake-efficient picoplankton could act as short-term, small-scale P storage under low P conditions, until PolyP is liberated again to support other phytoplankton in eutrophic waters. This community-scale resource allocation may therefore buffer seasonal P deficiencies, or from a management perspective, temporal restoration measures.

*Scenedesmus* does not show variation in its intracellular P concentrations under N limitation but does show an 8-fold lower P uptake rate (Rhee, 1974). Similar results were found for N-limited *Synechococcus* (Grillo and Gibson, 1979), pointing to an energy-saving mechanism of down-regulation of P uptake when N is limiting. Chl fluorescence is quenched after a P pulse delivered to P-limited cells of *Dunaliella tertiolecta* (Roberts *et al.*, 2008). This fluorescence signal could potentially be used as a monitoring tool to detect P limitation in cultures, and possibly in natural systems, if the fluorescence signal could be taxonomically resolved. A similar approach was tested in coastal waters by combining an HPLC with ChemTax and FRRf to track responses during the addition of either N or N + P to a natural phytoplankton assemblage (Zhao and Quigg, 2014). The approach allowed tracking of shifts in phytoplankton composition and therefore determination of groups competitive under pulsed nutrient supply.

Nutrient pulses are probably the rule in most systems, as phytoplankton cells experience pulse-wise exhaustion and resupply of nutrients within their diffusive boundary layer (Aubriot *et al.*, 2000). High uptake rates and nutrient-limited boundary layers around a cell can lead to a transport limitation, where the uptake is regulated by motion of the medium relative to the cell, and a diffusion quotient, rather than by bulk nutrient concentration (Pasciak and Gavis, 1974; Ploug *et al.*, 1999). Such diffusional limitation is influenced by cell size and, in larger cells, by motility or buoyancy control (Gemmell *et al.*, 2016). The determinations of physiological uptake rates may therefore differ from *in situ* uptake rates, depending on experimental setting, as for example mixing of the medium. The regulation of P uptake depends therefore on the timing of re-supply. If the amount of available external P does not drop after 15–25 minutes, cells down-regulate their uptake rates (Aubriot and Bonilla, 2012). However, down-regulation for genes for high-affinity P transporters only occurs if cells start to recover from P starvation (Solovchenko *et al.*, 2020). Such high-affinity P-uptake systems include the *sphX* gene in *Synechococcus* PCC7942, which enables cells to show pre-acclimated uptake kinetics to re-occurring P pulses, depending upon gene expression induced under earlier cycles of P pulses (Falkner *et al.*, 1998). These mechanisms enable some cells to exploit low, pulsed nutrient concentrations with high-affinity transporters, and thereby persist even when measures of bulk dissolved inorganic P are low. In contrast, a high background nutrient concentration will lead to expression of lower affinity systems to support a steady growth rate (Aubriot and Bonilla, 2012).

Monitoring external nutrient concentrations will not, therefore, on their own reveal nutrient limitations of the phytoplankton community. Monitoring nutrient concentrations needs to be complemented by knowledge of the physiological response capacity of the relevant phytoplankton assemblage, including capacities for luxury storage of nutrients (Hagemann *et al.*, 2019), recycling of organic nutrient sources (Labry *et al.*, 2005) or functional substitutions of nutrients (Van Mooy *et al.*, 2006) in cell compartments.

#### Silicate

In diatoms there are high- and low-affinity systems for Si, which in turn depend on intracellular binding components to incorporate Si (Thamatrakoln and Hildebrand, 2008). Those binding components are probably controlled within 5–10 minutes after intracellular Si-pools drop. The model diatom *Thalassiosira pseudonana* can sustain growth for three cell cycles without P addition, but ceases growing within 2 hours of silicate depletion (Parslow *et al.*, 1984). Furthermore, the timing of Si-depletion has an impact on how fast diatom cells stop and re-start growth. Diatoms of different species will experience a prolongation of their reproductive cycle (Brzezinski *et al.*, 1990; Martin-Jézéquel *et al.*, 2000), or even programmed cell death (Wang *et al.*, 2017), under Si-limiting conditions. This strong dependence of diatoms upon silicate generates striking ecological effects. For example, damming of the Danube River led to a 3-fold decrease of Si flux (Cociasu *et al.*, 1996), leading to a subsequent loss of diatoms from Black Sea surface waters (Humborg *et al.*, 1997). In any case, fluctuating or artificially lowered Si inputs in all aquatic systems will affect diatom blooms with subsequent effects on food webs. Lower Si content in diatoms makes them more prone to grazing (Liu *et al.*, 2016). Furthermore, the increase of atmospheric CO_2_ and lowered availability of Si in some waters may promote an increase of toxicity in *Pseudo-nitzschia fraudulenta* (Tatters *et al.*, 2012). Interestingly, the artificial addition of silicate can shift phytoplankton compositions from flagellates towards diatoms, albeit without increasing biogenic sedimentation rates, in the form of Chl a, in mesocosms (Svensen *et al.*, 2001). These results can be explained by onset of an additional Fe limitation, where Fe-deplete and Si-replete conditions will lead to thick-shelled diatoms (Assmy *et al.*, 2013), through prolonged Si-incorporation during Fe growth limitation (Wilken *et al.*, 2011). However, those cells will mostly sink as empty silicate shells, without promoting much C-transfer into deeper waters (Assmy *et al.*, 2013). The management option of controlling biogenic flows through forced species compositional changes may therefore depend upon co-limitations for other nutrients. Further work is thus needed to evaluate the feasibility of manipulating the Si cycle to prevent harmful blooms, or to manage food webs.

### Interactions of cell size with acclimation

Biovolume is not a fixed taxonomic trait but can rather change within phytoplankton taxa (Peter and Sommer, 2015; Bernstein *et al.*, 2016; Weisse *et al.*, 2016), either at single-cell level, or as a colony, thereby altering buoyancy control (Gemmell *et al.*, 2016). Cell size regulation can depend upon temperature, light, salinity, nutrient availability or grazer occurrence (Staehr *et al.*, 2002; Buma *et al.*, 2006; Staehr and Birkeland, 2006; Bernstein *et al.*, 2016; Ayache *et al.*, 2020; Lürling, 2020). Temperature is assumed to act directly on cell size, as increasing temperatures increase resource demands and solubility of CO_2_ in water decreases (Atkinson *et al.*, 2003).

Increasing temperatures may also increase growth rates of some taxa, thereby favouring small, fast-growing cells (Staehr and Birkeland, 2006). However, under optimized laboratory conditions, with no resource limitation, biovolume can increase at increasing temperatures (Thompson *et al.*, 1992). In contrast, high irradiance can increase cell size, with an increase of C content per cell in diatoms (Buma *et al.*, 2006), prasinophytes, chlorophytes (Staehr *et al.*, 2002) and cyanobacteria (Bernstein *et al.*, 2016). With increasing temperature and possibly light (through stratification) in the future, phytoplankton blooms may grow even faster in conjunction with higher available nutrients, thereby de-stabilizing and replacing current macrophyte-dominated systems.

Occurrence of grazers can promote cell size change (Lürling, 2020) within taxa or at the community level, towards either cells grown larger than digestible size (Lürling, 2003) or smaller towards passage through predator ingestion systems (Yoshida *et al.*, 2004). It is still debated how grazing will shape phytoplankton communities of the future, towards larger or smaller cells (reviewed in Sommer *et al.*, 2017), and trends will likely differ across systems.

## Population Resilience through Intraspecific Diversity

**Figure 3:**
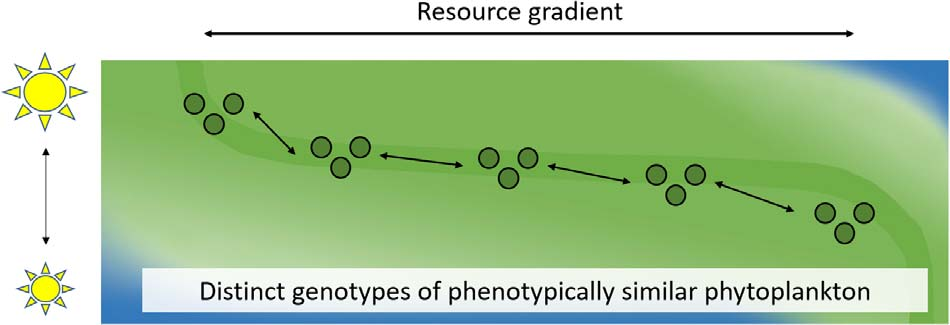
Hypothetical distribution of a sub-population across gradients of light and other resources. Depending on intraspecific diversity, genome size and encoded phenotypic plasticity, several genotypes can populate a wider range than one genotype alone. Shifts in resource availability will shift the relative share of a genotype within a sub-population.

Intraspecific interactions, with displacement of genotypes, can be recurrent seasonal successional replacements, with genotypes sequentially dominating, in contrast to genotype losses or replacements under sustained new environmental regimes (Fig. 3). Changing nutrients, light and temperature can act as bottlenecks, with only a sub-selection of genotypes surviving. Intraspecific genotype replacement has been found in planktonic cyanobacteria (Wilson *et al.*, 2006), diatoms (Godhe and Rynearson, 2017), green algae (Narwani *et al.*, 2015) and benthic red algae (Ursi *et al.*, 2003). Intraspecific replacements depend on the capacity of each genotype to cope with environmental changes through phenotypic plasticity (Callahan *et al.*, 2008) and their role within the ecosystem, whether C (competition) or S (survival) strategists based on either r or K selection (Reynolds, 2012). Indeed, a variety of genotypes can occur within an ecosystem without dominating it, as the metabolic acclimations or viral susceptibilities of each genotype may only allow net growth up to limitations imposed by environmental or biotic factors. Replacements of one genotype with a congener may have an ecological impact equivalent to loss of the taxon completely (Des Roches *et al.*, 2018) so losses of intraspecific variations may indirectly shape communities through, e.g. trophic cascades.

### Diversity-dependent niche width

The focus on a few selected genotypes as model organisms, including *Synechocystis* PCC6803 or *T. pseudonana* CCMP1335, may therefore be misleading when describing the wider physiological capacities of phytoplankton assemblages based upon phylogenetic composition (Kemp and Villareal, 2018) or broad shared traits, such as morphology. Gross morphology is not only a weak trait for phylogenetic relatedness (Alexandrou *et al.*, 2015; Albrecht *et al.*, 2017), but also a weak predictor of competitive advantage (Narwani *et al.*, 2015). Furthermore, describing phytoplankton taxa only by a particular mean trait value for an entire taxon may lead to an underestimation of the overall taxon physiological performance by up to one order of magnitude (Malerba *et al.*, 2016). Phytoplankton diversity changes sharply along gradients of, e.g. light (Urbach *et al.*, 1998) or ion composition (Laamanen *et al.*, 2002). Diversity is also likely driven by ecosystem perturbation rates, so that under stable conditions a specialist clade can dominate (Fuller *et al.*, 2003), whereas under constantly changing conditions, as in estuaries, higher diversity can persist (Chen *et al.*, 2006).

One prominent example for strong niche specialization is *Prochlorococcus* (Dufresne *et al.*, 2005), which constitutes, together with *Synechococcus*, 30–55% of carbon biomass of picophytoplankton in the oceans (Buitenhuis *et al.*, 2012). There are multiple *Prochlorococcus* ecotypes, with genome sizes varying from 1.6 to 2.6 MBp (Kettler *et al.*, 2007). These genotypes cannot replace each other in their respective niches. For example, MED4 as part of the HL-clade is adapted to high light and high O_2_ concentrations, whereas MIT9313 exploits low light (Rocap *et al.*, 2003). Closely related *Prochloroccocus* or *Synechococcus* genotypes (based on 16S rRNA) can differ in their genetic capacities for N and P uptake and utilization (Moore *et al.*, 2005; Martiny *et al.*, 2009a, 2009b), based on their encoded capacities to produce phosphatases, N or P uptake systems. Analogously, populations of the diatom *Ditylum brightwellii* differ between sampling locations in microsatellite allele distributions, but not in their 18S rDNA pattern (Rynearson and Armbrust, 2004).

### Effects on restoration

Analogous differences in genotype-specific responses occur in other groups including the chlorophytes *Ostreococcus* (Six *et al.*, 2012) and *Haematococcus* (Allewaert *et al.*, 2017)*,* the cyanobacteria *Microcystis* (Wilson *et al.*, 2006), the dinoflagellate *Alexandrium catenella* (Jauzein *et al.*, 2010) and the diatom *Thalassiosira hyalina* (Wolf *et al.*, 2018). For *Ostreococcus* one genotype might dominate under higher irradiances and elevated N inputs, whereas another genotype grows better under lower light and lower N concentrations (Six *et al.*, 2012). Two genotypes of the toxic dinoflagellate *A. catenella* showed different requirements for P, with regulation of uptake rate dependent on C:P ratios within cells (Jauzein *et al.*, 2010).

Such differences in P requirements can lead to bloom formations from genotypes needing less P, for example after nutrient inputs into a system have been lowered. Strongly lowering P favoured the occurrences of *Planktothrix* HABs in Lake Zurich (Posch *et al.*, 2012), whereas a non-toxic *Planktothrix* strain probably dominated during preceding eutrophic conditions (Ostermaier *et al.*, 2012). A similar high intraspecific variability was found across 32 isolates of *Microcystis aeruginosa* from 12 lakes based on an increase of population growth rate with colony biovolume (Wilson *et al.*, 2006). Five isolated genotypes of *M. aeruginosa* and *Raphidiopsis raciborskii* (Guedes *et al.*, 2019) showed either significantly different growth rates or different PSII efficiency at early stationary phases upon P depletion. However, pooling the effects within a species evened out the effects of each intra-species genotype, leading to no overall competitive advantage of one taxon over the other during P limitation. Thus, conserved phylogeny may not explain realized niches in phytoplankton, especially not when considering lateral gene transfer (Dufresne *et al.*, 2008) or sexual reproduction (Reusch and Boyd, 2013). From a management and conservation perspective, this intraspecific diversity is challenging, as widely applied morphological resolved data sets may not catch the actual trend of species and their respective physiological capacities during restoration. With the drop in cost of sequencing it is therefore useful to assess several genotypes per population to define potential and realized niches within a system. Information on community composition and presumed exploitative strategies will help decision makers to formulate individual options per ecosystem.

### Resistance and resilience

A question for future studies will be why a nuisance taxon does not rise to dominance even earlier during eutrophication. It is likely that the pre-eutrophication community itself showed resilience through genotype diversity, thereby resisting apparent succession. For example, disturbance through application of precipitating agents to control P (Barçante *et al.*, 2020) applies a pulse stress of nutrient withdrawal. Pulse-wise re-supply can then favour the fastest growing taxon, or intraspecific competitor (Klappenbach *et al.*, 2000), which rises to dominate a pulse-disturbed community. This exploitation of pulsed habitats can be positive for natural ecosystems experiencing disturbance, but also negative if such buffering capacities must be overcome to alter community composition towards a desired goal. In the case of a Baltic lagoon system, this stabilizes a perennial, morphotypically homogeneous but intraspecifically diverse phytoplankton assemblage (Schiewer, 2007; Albrecht *et al.*, 2017), by its exploitation of a pulse-wise nutrient supply for growth (Berthold and Schumann, 2020).

Instead of such a pulse-disturbance, a press-disturbance with sustained, strong lowering of nutrient inputs may suppress species rapidly exploiting pulsed nutrient supplies. Slow-growing species (low RNA/DNA ratio) make better use of systems with limiting nutrient concentrations, as they maximize resource use (Lee *et al.*, 2009). This strategy leads to an overall resource-limited ecosystem that is more resistant against press disturbance (Shade *et al.*, 2012). From the phytoplankton perspective, reducing nutrient inflows is a stress factor that is perceived as either press or pulse disturbance. Management actions need to consider not only the amplitude but also the frequency of restoration measures to overcome such variable community responses to resource limitation. Furthermore, half a century is sufficient to select for newly adapted strains during pre- and post-eutrophication, as seen from cyst revival experiments (Girault *et al.*, 2021) so there is no short return to a pre-disturbance array of genotypes.

Diversity can stabilize sub-populations of genotypes within and across habitat boundaries (Kremp *et al.*, 2016; Godhe and Rynearson, 2017). Multi-genotype populations of the Arctic diatom *T. hyalina* were able to buffer potential deviations from today’s temperature and pCO_2_ concentrations, through intraspecific selection for the best adapted among the genotypes (Wolf *et al.*, 2018, 2019). This genotype variation was identified as a driver of evolutionary and ecological success of diatoms, despite global change (reviewed in Godhe and Rynearson, 2017). Thus, genotype niches are smaller than the respective population niche, which leads to a population niche depending on both: local intraspecific diversity and the actual habitat (Fig. 3). Such population diversity has so far been described for freshwater and marine habitats (Tesson *et al.*, 2014; Van Den Wyngaert *et al.*, 2015). The rate of acclimation vs. adaptation in intraspecific diversity develops over several hundreds of generations to, for example temperature increase (Aranguren-Gassis *et al.*, 2019). With human management of inputs, the increase/decrease of key elements can shift the genotypic composition of a population, within similar morphotypes. For example, *Synechococcus* and *Synechocystis* share a similar phenotype, but show completely different transcriptional responses, when facing different abiotic conditions (Billis *et al.*, 2014). Such genetically diverse phytoplankton communities may contain several taxa with complementary response traits that have the potential for rapid compensatory growth after pulsed disturbance (Yachi and Loreau, 1999; Shade *et al.*, 2012).

## Conclusions and Prospects

Phytoplanktons thrive in all aquatic ecosystems worldwide, but human impacts shift the occurrences and frequencies of taxa. The management of aquatic systems needs to consider not only nutrient inputs and balances, but also the microdiversity of phytoplankton. Phytoplankton responses are not fixed but depend upon the genetic diversity that underlies physiological diversity, which in turn drives the functional diversity within an ecosystem. Unravelling the hidden diversity and ranges of physiological responses, on an intrataxa as well as intertaxa level is a next step towards understanding phytoplankton succession. However, all these patterns depend on knowledge from the bottom (nutrients, light, temperature) and the top (higher trophic levels). For that, long-term monitoring data sets continue to be necessary, to inform and define the needed ecophysiological and genetic studies.

### A Baltic case study

A prediction into the future is difficult, yet some systems, as for example the Baltic Sea, can be used as models (Reusch *et al.*, 2018) due to above-average temperature increase and a long history of monitored eutrophication, with its densely inhabited catchment area and low water exchange with the Atlantic Ocean. Policy interventions to control and reverse eutrophication are now well advanced in the Baltic region, temporally overlapping with surface water warming in recent decades (HELCOM, 2014).

In the Baltic Sea the seasonal Chl-maximum has already shifted from a diatom bloom in spring towards the cyanobacteria bloom in summer within four decades (Kahru *et al.*, 2016). This increase, extension and shift of dominant blooming taxa is permitted by non-limiting nutrients in spring (Fisher *et al.*, 1992; Pastuszak *et al.*, 2003) earlier onset of a high cumulative amount of incoming shortwave irradiance through altered cloudiness patterns (Kahru *et al.*, 2016) or by altered hydrological flows into the system through either heavy rainfall (Mallin *et al.*, 1993) or exchange with the open sea (Trombetta *et al.*, 2019). More broadly, water temperature also acts through complex interactions with the abiotic environment and other trophic levels. Water temperature regulates turn-over times of nutrients within the water column, by altering re-mineralization within the sediment and water column and through altering nutrient transport rates throughout the water column by increased stratification (Behrenfeld *et al.*, 2006; Rykaczewski and Dunne, 2010; Kraemer *et al.*, 2015; Kemp and Villareal, 2018). For example, increasing water temperatures increase oxygen consumption by sediments, lowering the redox poise of sediments and therefore mediating nutrient releases, particularly phosphate, which can (re)fuel primary production (Cowan and Boynton, 1996; Spears *et al.*, 2008).

This increased nutrient background will further shape phytoplankton succession by favouring growth strategies depending on frequency or spatial distribution of nutrients. A regulated drop in external nutrient supply can lead to a compositional change towards taxa that are able to control their buoyancy and take nutrients up from the thermocline, as described for lakes and the Baltic Sea (Nausch *et al.*, 2012; Posch *et al.*, 2012). However, non-point sources from the coast may still influence the Baltic Proper through large-scale eddy-mediated transport of plankton and nutrients (Vortmeyer-Kley *et al.*, 2019).

The Baltic Sea and its tributaries show resistance to reversion of past eutrophication, with current dominance by a large diversity of previously unrecognized smaller phytoplankters in the Baltic and its coastal basins (Hugerth *et al*., 2015; Albrecht *et al*., 2017; Celepli *et al*., 2017). These small phytoplankters are possibly favoured by lowered, pulsewise nutrient releases and increasing temperatures. In lagoon systems of the Baltic Sea, with negligible nutrient input from the suboxic waters/sediments, phytoplankton grows adjacent to the wetland, but not in the centre of the water body (Berthold *et al*., 2018). In such shallow waters, nutrient runoff from land (Karstens *et al*., 2016) and from fluctuating redox conditions within the wetland (Karstens *et al*., 2015), can deliver P pulse wise to the phytoplankton, which is not light limited in the shallow waters.

The combination of increasing temperature and decreasing nutrients in input-managed systems can lower the overall biovolume of phytoplankton communities (Peter and Sommer, 2013), potentially leading to dominance of smaller cells in future warmer aquatic ecosystems (Daufresne *et al.*, 2009; Sommer *et al.*, 2017). As mentioned above, small cell diameter can be an adaptation to low light (Vincent, 2000), particularly in conjunction with low nutrients that place a premium upon light capture per invested elemental resource (Finkel, 2007). Cell size strongly influences nutrient uptake (Finkel *et al.*, 2004) and cell quotas for key elements (Finkel *et al.*, 2010a). Establishment of a phytoplankton community dominated by small cells may therefore pre-empt the re-establishment of larger phytoplankters, or macrophytes, through shading and through rapid re-uptake of recycled nutrients. Systems with such increasing dominance by picophytoplankton have been described for freshwater, brackish and shelf sea areas (Schiewer, 2007; Callieri, 2008; Schmidt *et al.*, 2020), when nutrients and light become limiting to other groups.

In contrast, large cyanobacteria tend to dominate, when the overall turbidity, temperature and TP concentration is high (Scheffer *et al.*, 1997; Håkanson *et al.*, 2007; Schwaderer *et al.*, 2011). Thin filaments of larger cyanobacteria capture light in turbid waters as well as the same biovolume of small cells (Kirk, 1976). Larger colonies alter microscale oxygen environments, favouring N fixation (Ploug, 2008; Stal, 2017), but even single filaments of *Trichodesmum* can show higher N fixation rates than colonies (Eichner *et al.*, 2019). There may be other advantages of colony formation besides N fixation. Thus, the postulated dominance of small cells may not become a universal pattern. Ecosystems will eventually change with the food webs adapting to the prevailing composition of primary producers (Motwani and Gorokhova, 2013; Liénart *et al.*, 2021), thereby affecting higher ecosystem services. If a shift towards toxic blooms or picophytoplankton dominance alters ecosystem services, managing systems towards new or renewed communities requires predictive understanding of how these phytoplankton communities persist in exploiting post-eutrophied systems (Table 1). And, on a cautionary note, multiple nuisance taxa may well replace each other before some desired ecosystem state is reached.

## References

[ref1] Albrecht M, Pröschold T, Schumann R (2017) Identification of cyanobacteria in a eutrophic coastal lagoon on the Southern Baltic Coast. Front Microbiol 8: 1–16. 28611738 10.3389/fmicb.2017.00923 PMC5446986

[ref2] Alexandrou MA, Cardinale BJ, Hall JD, Delwiche CF, Fritschie K, Narwani A, Venail PA, Bentlage B, Pankey MS, Oakley TH (2015) Evolutionary relatedness does not predict competition and co-occurrence in natural or experimental communities of green algae. Proc R Soc B Biol Sci 282: 20141745. 10.1098/rspb.2014.1745 PMC4286037 25473009

[ref3] Allesson L, Andersen T, Dörsch P, Eiler A, Wei J, Hessen DO (2020) Phosphorus availability promotes bacterial DOC-mineralization, but not cumulative CO2-production. Front Microbiol 11. 10.3389/fmicb.2020.569879. PMC7541949 33072029

[ref4] Allewaert CC, Vanormelingen P, Daveloose I, Verstraete T, Vyverman W (2017) Intraspecific trait variation affecting astaxanthin productivity in two Haematococcus (Chlorophyceae) species. Algal Res 21: 191–202.

[ref5] Allison SD, Martiny JBH (2009) Resistance, resilience, and redundancy in microbial communities. Proc Natl Acad Sci 2: 149–166. 10.1073/pnas.0801925105 PMC2556421 18695234

[ref6] Andersen IM, Williamson TJ, González MJ, Vanni MJ (2020) Nitrate, ammonium, and phosphorus drive seasonal nutrient limitation of chlorophytes, cyanobacteria, and diatoms in a hyper-eutrophic reservoir. Limnol Oceanogr 65: 962–978.

[ref7] Aranguren-Gassis M, Kremer CT, Klausmeier CA, Litchman E (2019) Nitrogen limitation inhibits marine diatom adaptation to high temperatures. Ecol Lett 22: 1860–1869. 31429516 10.1111/ele.13378

[ref8] Asmala E, Osburn CL, Paerl RW, Paerl HW (2021) Elevated organic carbon pulses persist in estuarine environment after major storm events. Limnol Oceanogr Lett 6: 43–50.

[ref9] Assmy P, Smetacek V, Montresor M, Klaas C, Henjes J, Strass VH, Arrieta JM, Bathmann U, Berg GM, Breitbarth E et al. (2013) Thick-shelled, grazer-protected diatoms decouple ocean carbon and silicon cycles in the iron-limited Antarctic Circumpolar Current. Proc Natl Acad Sci U S A 110: 20633–20638. 24248337 10.1073/pnas.1309345110 PMC3870680

[ref10] Atkinson D, Ciotti BJ, DJS M (2003) Protists decrease in size linearly with temperature: ca. 2.5%°C -1. Proc R Soc B Biol Sci 270: 2605–2611. 10.1098/rspb.2003.2538 PMC1691543 14728784

[ref11] Aubriot L, Bonilla S (2012) Rapid regulation of phosphate uptake in freshwater cyanobacterial blooms. Aquat Microb Ecol 67: 251–263.

[ref12] Aubriot L, Wagner F, Falkner G (2000) The phosphate uptake behaviour of phytoplankton communities in eutrophic lakes reflects alterations in the phosphate supply. Eur J Phycol 35: 255–262.

[ref13] Ayache N, Hervé F, Lundholm N, Amzil Z, Caruana AMN (2020) Acclimation of the marine diatom pseudo-nitzschia australis to different salinity conditions: effects on growth, photosynthetic activity, and domoic acid content. J Phycol 56: 97–109. 31591715 10.1111/jpy.12929

[ref14] Azam F, Fenchel T, Field JG, Gray JS, Meyer-Reil LA, Thingstad F (1983) The ecological role of water-column microbes in the sea. Mar Ecol Prog Ser 10: 257–263.

[ref15] Bailey S, Grossman A (2008) Photoprotection in cyanobacteria: regulation of light harvesting. Photochem Photobiol 84: 1410–1420. 19067963 10.1111/j.1751-1097.2008.00453.x

[ref16] Barçante B, Nascimento NO, Silva TFG, Reis LA, Giani A (2020) Cyanobacteria dynamics and phytoplankton species richness as a measure of waterbody recovery: response to phosphorus removal treatment in a tropical eutrophic reservoir. Ecol Indic 117: 106702.

[ref17] Barker-Aström K, Schelin J, Gustafsson P, Clarke AK, Campbell DA (2005) Chlorosis during nitrogen starvation is altered by carbon dioxide and temperature status and is mediated by the ClpP1 protease in *Synechococcus elongatus*. Arch Microbiol 183: 66–69. 15549267 10.1007/s00203-004-0741-x

[ref18] Barton AD, Irwin AJ, Finkel ZV, Stock CA (2016) Anthropogenic climate change drives shift and shuffle in North Atlantic phytoplankton communities. Proc Natl Acad Sci U S A 113: 2964–2969. 26903635 10.1073/pnas.1519080113 PMC4801288

[ref19] Behrenfeld MJ, Maran E, Siegel DA, Hooker SB (2002) Photoacclimation and nutrient-based model of light-saturated photosynthesis for quantifying oceanic primary production. Mar Ecol Prog Ser 228: 103–117.

[ref20] Behrenfeld MJ, O’Malley RT, Siegel DA, McClain CR, Sarmiento JL, Feldman GC, Milligan AJ, Falkowski PG, Letelier RM, Boss ES (2006) Climate-driven trends in contemporary ocean productivity. Nature 444: 752–755. 17151666 10.1038/nature05317

[ref21] Berges JA, Mulholland MR (2008) Enzymes and nitrogen cycling. In Nitrogen in the Marine Environment. Elsevier, London, pp 1385–1444.

[ref22] Bernstein HC, McClure RS, Hill EA, Markillie LM, Chrisler WB, Romine MF, McDermott JE, Posewitz MC, Bryant DA, Konopka AE et al. (2016) Unlocking the constraints of cyanobacterial productivity: acclimations enabling ultrafast growth. MBio 7: 1–10. 10.1128/mBio.00949-16 PMC4981716 27460798

[ref23] Berthold M, Karstens S, Buczko U, Schumann R (2018) Potential export of soluble reactive phosphorus from a coastal wetland in a cold-temperate lagoon system: buffer capacities of macrophytes and impact on phytoplankton. Sci Total Environ 616–617: 46–54. 10.1016/j.scitotenv.2017.10.244 29107778

[ref24] Berthold M, Nausch G, Weber MV, Koch S, Kahle P, Lennartz B, Tränckner J, Buczko U, Tonn C, Ekardt F et al. (2019a) Phosphorus and the Baltic Sea: sustainable management. In P Maurice, ed, Encyclopedia of Water: Science, Technology, and Society, Ed 1st. Wiley-VCH, pp. 2479–2498.

[ref25] Berthold M, Paar M (2021) Dynamics of primary productivity in relation to submerged vegetation of a shallow, eutrophic lagoon : a field and mesocosm study. PLoS One 15: e0247696. 10.1371/journal.pone.0247696 PMC8101763 33956797

[ref26] Berthold M, Schumann R (2020) Phosphorus dynamics in a eutrophic lagoon: uptake and utilization of nutrient pulses by phytoplankton. Front Mar Sci 7: 1–15.32802822

[ref27] Berthold M, Wulff R, Reiff V, Karsten U, Nausch G, Schumann R (2019b) Magnitude and influence of atmospheric phosphorus deposition on the southern Baltic Sea coast over 23 years: implications for coastal waters. Environ Sci Eur 31: 27.

[ref28] Billis K, Billini M, Tripp HJ, Kyrpides NC, Mavromatis K (2014) Comparative transcriptomics between Synechococcus PCC 7942 and Synechocystis PCC 6803 provide insights into mechanisms of stress acclimation. PLoS One 9: e109738. 25340743 10.1371/journal.pone.0109738 PMC4207680

[ref29] Bissinger JE, Montagnes DJS, Sharples J, Atkinson D (2008) Predicting marine phytoplankton maximum growth rates from temperature: improving on the Eppley curve using quantile regression. Limnol Oceanogr 53: 487–493.

[ref30] Blindow I, Andersson G, Hargeby A (1993) Long-term pattern of alternative stable states in two shallow eutrophic lakes. Freshw Biol 30: 159–167.

[ref31] Bonisteel EM, Turner BE, Murphy CD, Melanson J-R, Duff NM, Beardsall BD, Xu K, Campbell DA, Cockshutt AM (2018) Strain specific differences in rates of photosystem II repair in picocyanobacteria correlate to differences in FtsH protein levels and isoform expression patterns. PLoS One 13: e0209115. 30566504 10.1371/journal.pone.0209115 PMC6300248

[ref32] Boyd PW, Rynearson TA, Armstrong EA, Fu F, Hayashi K, Hu Z, Hutchins DA, Kudela RM, Litchman E, Mulholland MR et al. (2013) Marine phytoplankton temperature versus growth responses from polar to tropical waters - outcome of a scientific community-wide study. PLoS One 8: e63091. 23704890 10.1371/journal.pone.0063091 PMC3660375

[ref33] Broecker WS, Henderson GM (1998) The sequence of events surrounding Termination II and their implications for the cause of glacial-interglacial CO2 changes. Paleoceanography 13: 352–364.

[ref34] Brzezinski M, Olson R, Chisholm S (1990) Silicon availability and cell-cycle progression in marine diatoms. Mar Ecol Prog Ser 67: 83–96.

[ref35] Buitenhuis ET, Li WKW, Vaulot D, Lomas MW, Landry MR, Partensky F, Karl DM, Ulloa O, Campbell L, Jacquet S et al. (2012) Picophytoplankton biomass distribution in the global ocean. Earth Syst Sci Data 4: 37–46.

[ref36] Buma AGJ, Wright SW, Van Den R, Van De WH, Davidson AT (2006) PAR acclimation and UVBR-induced DNA damage in Antarctic marine microalgae. Mar Ecol Prog Ser 315: 33–42.

[ref37] Burke L, Kura Y, Kassem K, Revenga C, Spalding M, McAllister D (2001) Pilot Analysis of Global Ecosystems: Coastal Ecosystems. Coastal Ecosystems. World Resources Institute, Washington D.C., pp. 73–78.

[ref38] Callahan HS, Maughan H, Steiner UK (2008) Phenotypic plasticity, costs of phenotypes, and costs of plasticity: toward an integrative view. Ann N Y Acad Sci 1133: 44–66. 18559815 10.1196/annals.1438.008

[ref39] Callieri C (2008) Picophytoplankton in freshwater ecosystems: the importance of small-sized phototrophs. Freshw Rev 1: 1–28.

[ref40] Campbell DA, Hossain Z, Cockshutt AM, Zhaxybayeva O, Wu H, Li G (2013) Photosystem II protein clearance and FtsH function in the diatom *Thalassiosira pseudonana*. Photosynth Res 115: 43–54. 23504483 10.1007/s11120-013-9809-2

[ref41] Campbell DA, Serôdio J (2020) Photoinhibition of Photosystem II in phytoplankton: processes and patterns. In AWD Larkum, AR Grossmann, JA Raven, eds, Photosynthesis in Algae: Biochemical and Physiological Mechanisms. Springer International Publishing, Cham, pp. 329–365.

[ref325] Celepli N, Sundh J, Ekman M, Dupont CL, Yooseph S, Bergman B, Ininbergs K (2017) Meta-omic analyses of Baltic Sea cyanobacteria: diversity, community structure and salt acclimation. Environ Microbiol 19: 673–686. 27871145 10.1111/1462-2920.13592

[ref42] Chen F, Wang K, Kan J, Suzuki MT, Wommack KE (2006) Diverse and unique picocyanobacteria in Chesapeake Bay, revealed by 16S-23S rRNA internal transcribed spacer sequences. Appl Environ Microbiol 72: 2239–2243. 16517680 10.1128/AEM.72.3.2239-2243.2006 PMC1393199

[ref43] Chislock MF, Sharp KL, Wilson AE (2014) Cylindrospermopsis raciborskii dominates under very low and high nitrogen-to-phosphorus ratios. Water Res 49: 207–214. 24333522 10.1016/j.watres.2013.11.022

[ref44] Chorus I, Köhler A, Beulker C, Fastner J, van de K, Hegewald T, Hupfer M (2020) Decades needed for ecosystem components to respond to a sharp and drastic phosphorus load reduction. Hydrobiologia 847: 4621–4651.

[ref45] Chorus I, Spijkerman E (2021) What Colin Reynolds could tell us about nutrient limitation, N:P ratios and eutrophication control. Hydrobiologia 848: 95–111.

[ref46] Cloern JE (1996) Phytoplankton bloom dynamics in coastal ecosystems: a review with some general lessons from sustained investigation of San Francisco Bay, California. Rev Geophys 34: 127–168.

[ref47] Cloern JE (2018) Why large cells dominate estuarine phytoplankton. Limnol Oceanogr 63: S392–S409.

[ref48] Cociasu A, Dorogan L, Humborg C, Popa L (1996) Long-term ecological changes in Romanian coastal waters of the Black Sea. Mar Pollut Bull 32: 32–38.

[ref49] Conley DJ, Paerl HW, Howarth RW, Boesch DF, Seitzinger SP, Havens KE, Lancelot C, Likens GE (2009) Ecology-controlling eutrophication: nitrogen and phosphorus. Science 323: 1014–1015. 19229022 10.1126/science.1167755

[ref50] Contreras-Rosales LA, Schefuß E, Meyer V, Palamenghi L, Lückge A, Jennerjahn TC (2016) Origin and fate of sedimentary organic matter in the northern Bay of Bengal during the last 18 ka. Glob Planet Change 146: 53–66.

[ref51] Cowan J, Boynton W (1996) Sediment-water oxygen and nutrient exchanges along the longitudinal axis of Chesapeake Bay: seasonal patterns, controlling factors and ecological significance. Estuaries 19: 562–580.

[ref52] Crutzen PJ (2002) Geology of mankind. Nature 415: 23. 11780095 10.1038/415023a

[ref53] Dam BRV, Tobias C, Holbach A, Paerl HW, Zhu G (2018) CO2 limited conditions favor cyanobacteria in a hypereutrophic lake: an empirical and theoretical stable isotope study. Limnol Oceanogr 63: 1643–1659.

[ref54] Daufresne M, Lengfellner K, Sommer U (2009) Global warming benefits the small in aquatic ecosystems. Proc Natl Acad Sci 106: 12788–12793. 19620720 10.1073/pnas.0902080106 PMC2722360

[ref55] de VN, Elliott M, Orive E (2002) Causes, historical development, effects and future challenges of a common environmental problem: eutrophication. Hydrobiologia 475–476: 1–19.

[ref56] de Mazancourt C, Schwartz MW (2012) Starve a competitor: evolution of luxury consumption as a competitive strategy. Theor Ecol 5: 37–49.

[ref57] Des Roches S, Post DM, Turley NE, Bailey JK, Hendry AP, Kinnison MT, Schweitzer JA, Palkovacs EP (2018) The ecological importance of intraspecific variation. Nat Ecol Evol 2: 57–64. 29203921 10.1038/s41559-017-0402-5

[ref58] Deutsch B, Forster S, Wilhelm M, Dippner JW, Voss M (2010) Denitrification in sediments as a major nitrogen sink in the Baltic Sea: an extrapolation using sediment characteristics. Biogeosciences 7: 3259–3271.

[ref59] Diaz JM, Steffen R, Sanders JG, Tang Y, Duhamel S (2019) Preferential utilization of inorganic polyphosphate over other bioavailable phosphorus sources by the model diatoms \textlessi\textgreaterThalassiosira\textless/i\textgreater spp. Environ Microbiol 21: 2415–2425. 30972877 10.1111/1462-2920.14630 PMC6849833

[ref60] Dortch Q (1990) The interaction between ammonium and nitrate uptake in phytoplankton. Mar Ecol Prog Ser 61: 183–201.

[ref61] Droop MRM (1973) Some thoughts on nutrient limitation in algae. J Phycol 9: 264–272.

[ref62] Duarte CM, Conley DJ, Carstensen J, Sánchez-Camacho M (2009) Return to Neverland: shifting baselines affect eutrophication restoration targets. Estuaries Coasts 32: 29–36.

[ref63] Dufresne A, Garczarek L, Partensky F (2005) Accelerated evolution associated with genome reduction in a free-living prokaryote. Genome Biol 6: 1–10. 10.1186/gb-2005-6-2-r14 PMC551534 15693943

[ref64] Dufresne A, Ostrowski M, Scanlan DJ, Garczarek L, Mazard S, Palenik BP, Paulsen IT, de NT, Wincker P, Dossat C et al. (2008) Unraveling the genomic mosaic of a ubiquitous genus of marine cyanobacteria. Genome Biol 9: R90. 18507822 10.1186/gb-2008-9-5-r90 PMC2441476

[ref65] Dyhrman ST, Palenik B (1999) Phosphate stress in cultures and field populations of the dinoflagellate Prorocentrum minimum detected by a single-cell alkaline phosphate assay. Appl Environ Microbiol 65: 3205–3212. 10388722 10.1128/aem.65.7.3205-3212.1999 PMC91475

[ref66] Edwards KF, Thomas MK, Klausmeier CA, Litchman E (2016) Phytoplankton growth and the interaction of light and temperature: a synthesis at the species and community level. Limnol Oceanogr 61: 1232–1244.

[ref67] Eichner M, Thoms S, Rost B, Mohr W, Ahmerkamp S, Ploug H, Kuypers MMM, de D (2019) N2 fixation in free-floating filaments of Trichodesmium is higher than in transiently suboxic colony microenvironments. New Phytol 222: 852–863. 30507001 10.1111/nph.15621 PMC6590460

[ref68] Ellis M, Trachtenberg Z (2014) Which Anthropocene is it to be? Beyond geology to amoral and public discourse. Earths Future 2: 122–125.

[ref69] Elser JJ, Marzolf ER, Goldman CR (1990) Phosphorus and nitrogen limitation of phytopankton growth in the freshwaters of North America: a review and critique of experiments enrichments. Can J Fish Aquat Sci 47: 1468–1477.

[ref70] Elster H-J (1958) Lake classification, production and consumption. Int Ver Für Theor Angew Limnol Verhandlungen 13: 101–120.

[ref71] Eppley RW (1972) Temperature and phytoplankton growth in the sea. Fish Bull 70: 1063–1085.

[ref72] Falkner R, Wagner F, Aiba H, Falkner G (1998) Phosphate-uptake behaviour of a mutant of Synechococcus sp. PCC 7942 lacking one protein of the high-affinity phosphate-uptake system. Planta 206: 461–465.

[ref73] Falkowski P, Scholes RJ, Boyle E, Canadell J, Canfield D, Elser J, Gruber N, Hibbard K, Hogberg P, Linder S et al. (2000) The global carbon cycle: a test of our knowledge of earth as a system. Science 290: 291–296. 11030643 10.1126/science.290.5490.291

[ref74] Falkowski PG, Owens TG (1980) Light–shade adaptation 1. Plant Physiol 66: 592–595. 16661484 10.1104/pp.66.4.592 PMC440685

[ref75] Falkowski PG, Owens TG, Ley AC, Mauzerall DC (1981) Effects of growth irradiance levels on the ratio of reaction centers in two species of marine phytoplankton 1. Plant Physiol 68: 969–973. 16662035 10.1104/pp.68.4.969 PMC426022

[ref76] Fan C, Glibert PM, Burkholder JAM (2003) Characterization of the affinity for nitrogen, uptake kinetics, and environmental relationships for Prorocentrum minimum in natural blooms and laboratory cultures. Harmful Algae 2: 283–299.

[ref77] Ferber LR, Levine SN, Lini A, Livingston GP (2004) Do cyanobacteria dominate in eutrophic lakes because they fix atmospheric nitrogen? Freshw Biol 49: 690–708.

[ref78] Finkel Z (2001) Light absorption and size scaling of light-limited metabolism in marine diatoms. Limnol Oceanogr 46: 86–94.

[ref79] Finkel ZV (2007) Does phytoplankton size matter? The evolution of modern marine food webs. In PG Falkowski, AH Knoll, eds, Evolution of Primary Producers in the Sea. Elsevier Academic Press, Amsterdam ; Boston, pp. 334–345.

[ref80] Finkel ZV, Beardall J, Flynn KJ, Quigg A, Rees TAV, Raven JA (2010a) Phytoplankton in a changing world: cell size and elemental stoichiometry. J Plankton Res 32: 119–137.

[ref81] Finkel ZV, Beardall J, Flynn KJ, Quigg A, Rees TAV, Raven JA (2010b) Phytoplankton in a changing world: cell size and elemental stoichiometry. J Plankton Res 32: 119–137.

[ref82] Finkel ZV, Irwin AJ, Schofield O (2004) Resource limitation alters the 3/4 size scaling of metabolic rates in phytoplankton. Mar Ecol Prog Ser 273: 269–279.

[ref83] Fisher TR, Peele ER, Ammerman JW, Harding LW (1992) Nutrient limitation of phytoplankton in Chesapeake Bay. Mar Ecol Prog Ser 82: 51–63.

[ref84] Fogel ML, Ciiuentes LA, Velinsky DJ, Sharp JH (1992) Relationship of carbon availability in estuarine phytoplankton to isotopic composition. Mar Ecol Prog Ser 82: 291–300.

[ref85] Fonseca MS, Kenworthy WJ, Courtney FX, Hall MO (1994) Seagrass planting in the Southeastern United States: methods for accelerating habitat development. Restor Ecol 2: 198–212.

[ref86] Fuller NJ, Marie D, Vaulot D, Post AF, Scanlan DJ (2003) Clade-specific 16S ribosomal DNA oligonucleotides reveal the predominance of a single marine Synechococcus clade throughout a stratified water column in the Red Sea. Appl Environ Microbiol 69: 2430–2443. 12732508 10.1128/AEM.69.5.2430-2443.2003 PMC154553

[ref87] Gao K, Campbell DA (2014) Photophysiological responses of marine diatoms to elevated CO_2_ and decreased pH: a review. Funct Plant Biol 41: 449. 32481004 10.1071/FP13247

[ref88] Gao K, Helbling EW, Hder D, Hutchins DA (2012) Responses of marine primary producers to interactions between ocean acidification, solar radiation, and warming. Mar Ecol Prog Ser 470: 167–189.

[ref89] Garrido M, Cecchi P, Collos Y, Agostini S, Pasqualini V (2016) Water flux management and phytoplankton communities in a Mediterranean coastal lagoon. Part I: how to promote dinoflagellate dominance? Mar Pollut Bull 104: 139–152. 26869094 10.1016/j.marpolbul.2016.01.049

[ref90] Geider RJ (1987) Light and temperature dependence of the carbon to chlorophyll a ratio in microalgae and cyanobacteria: implications for physiology and growth of phytoplankton. New Phytol 106: 1–34.

[ref91] Geider RJ, La J (2002) Redfield revisited: variability of C:N:P in marine microalgae and its biochemical basis. Eur J Phycol 37: 1–17.

[ref92] Geiß U, Selig U, Schumann R, Steinbruch R, Bastrop R, Hagemann M, Schoor A (2004) Investigations on cyanobacterial diversity in a shallow estuary (Southern Baltic Sea) including genes relevant to salinity resistance and iron starvation acclimation. Environ Microbiol 6: 377–387. 15008815 10.1111/j.1462-2920.2004.00569.x

[ref93] Gemmell BJ, Oh G, Buskey EJ, Villareal TA (2016) Dynamic sinking behaviour in marine phytoplankton: rapid changes in buoyancy may aid in nutrient uptake. Proc R Soc B Biol Sci 283: 20161126. 10.1098/rspb.2016.1126 PMC5069504 27708154

[ref94] Ger KA, Naus-Wiezer S, De Meester L, Lürling M (2019) Zooplankton grazing selectivity regulates herbivory and dominance of toxic phytoplankton over multiple prey generations. Limnol Oceanogr 64: 1214–1227.

[ref95] Gessner F (1935) Phosphat und nitrat als produktionsfaktoren der gewässer. Int Ver Für Theor Angew Limnol Verhandlungen 7: 525–538.

[ref96] Girault M, Siano R, Labry C, Latimier M, Jauzein C, Beneyton T, Buisson L, Del Y, Baret J-C (2021) Variable inter and intraspecies alkaline phosphatase activity within single cells of revived dinoflagellates. ISME J 15: 2057–2069. 33568788 10.1038/s41396-021-00904-2 PMC8245567

[ref97] Glibert PM, Wilkerson FP, Dugdale RC, Parker AE, Alexander J, Blaser S, Murasko S (2014) Phytoplankton communities from San Francisco Bay Delta respond differently to oxidized and reduced nitrogen substrates-even under conditions that would otherwise suggest nitrogen sufficiency. Front Mar Sci 1: 1–16.

[ref98] Glibert PM, Wilkerson FP, Dugdale RC, Raven JA, Dupont CL, Leavitt PR, Parker AE, Burkholder JM, Kana TM (2016) Pluses and minuses of ammonium and nitrate uptake and assimilation by phytoplankton and implications for productivity and community composition, with emphasis on nitrogen-enriched conditions. Limnol Oceanogr 61: 165–197.

[ref99] Godhe A, Rynearson T (2017) The role of intraspecific variation in the ecological and evolutionary success of diatoms in changing environments. Philos Trans R Soc B Biol Sci 372: 20160399. 10.1098/rstb.2016.0399 PMC5516108 28717025

[ref100] González-Gil S, Keafer BA, Jovine RVM, Aguilera A, Lu S, Anderson DM (1998) Detection and quantification of alkaline phosphatase in single cells of phosphorus-starved marine phytoplankton. Mar Ecol Prog Ser 164: 21–35.

[ref101] Graff JR, Westberry TK, Milligan AJ, Brown MB, Dall’Olmo G, Reifel KM, Behrenfeld MJ (2016) Photoacclimation of natural phytoplankton communities. Mar Ecol Prog Ser 542: 51–62.

[ref102] Grébert T, Doré H, Partensky F, Farrant G, Boss E, Picheral M, Guidi L, Pesant S, Scanlan D, Wincker P et al. (2018) Light color acclimation is a key process in the global ocean distribution of Synechococcus cyanobacteria. Proc Natl Acad Sci 115: E2010–E2019. 29440402 10.1073/pnas.1717069115 PMC5834698

[ref103] Grillo JF, Gibson J (1979) Regulation of phosphate accumulation in the unicellular cyanobacterium Synechococcus. J Bacteriol 140: 508–517. 227842 10.1128/jb.140.2.508-517.1979 PMC216676

[ref329] Grover JP (1989) Phosphorus-dependent growth kinetics of 11 species of freshwater algae. Limnol Oceanogr 34: 341–348.

[ref104] Grover JP, Scott JT, Roelke DL, Brooks BW (2020) Dynamics of nitrogen-fixing cyanobacteria with heterocysts: a stoichiometric model. Mar Freshw Res 71: 644–658.

[ref105] Gruber N, Sarmiento J (1997) Global patterns of marine nitrogen fixation and denitrification. Global Biogeochem Cycles 11: 235.

[ref106] Guedes IA, Pacheco ABF, Vilar MCP, Mello MM, Marinho MM, Lurling M, Azevedo SMFO (2019) Intraspecific variability in response to phosphorus depleted conditions in the cyanobacteria Microcystis aeruginosa and Raphidiopsis raciborskii. Harmful Algae 86: 96–105. 31358281 10.1016/j.hal.2019.03.006

[ref107] Gulati RD, van E (2002) Lakes in the Netherlands, their origin, eutrophication and restoration: state-of-the-art review. In PH Nienhuis, RD Gulati, eds, Ecological Restoration of Aquatic and Semi-Aquatic Ecosystems in the Netherlands (NW Europe). Springer, Netherlands, Dordrecht, pp. 73–106.

[ref108] Gunduz B, Aydin F, Aydin I, Hamamci C (2011) Study of phosphorus distribution in coastal surface sediment by sequential extraction procedure (NE Mediterranean Sea, Antalya-Turkey). Microchem J 98: 72–76.

[ref109] Gunnars A, Blomqvist S, Johansson P, Andersson C (2002) Formation of Fe (III) oxyhydroxide colloids in freshwater and brackish seawater, with incorporation of phosphate and calcium. Geochim Cosmochim Acta 66: 745–758.

[ref110] Hagemann M, Möke F, Springer A, Westermann L, Frank M, Wasmund N, Bauwe H (2019) Cyanobacterium Nodularia spumigena strain CCY9414 accumulates polyphosphate under long-term P-limiting conditions. Aquat Microb Ecol 82: 265–274.

[ref111] Håkanson L, Bryhn AAC, Hytteborn JKJ (2007) On the issue of limiting nutrient and predictions of cyanobacteria in aquatic systems. Sci Total Environ 379: 89–108. 17448525 10.1016/j.scitotenv.2007.03.009

[ref112] Hansen PJ, Lundholm N, Rost B (2007) Growth limitation in marine red-tide dinoflagellates: efects of pH versus inorganic carbon availability. Mar Ecol Prog Ser 334: 10.

[ref113] Härnström K, Ellegaard M, Andersen TJ, Godhe A (2011) Hundred years of genetic structure in a sediment revived diatom population. Proc Natl Acad Sci 108: 4252–4257. 21282612 10.1073/pnas.1013528108 PMC3054006

[ref114] Hasler AD (1947) Eutrophication of lakes by domestic drainage. Ecology 28: 383–395.

[ref115] HELCOM (2014) Eutrophication status of the Baltic Sea 2007-2011—a concise thematic assessment. Balt Sea Environ Proc 143: 25.

[ref116] Hessen DO, Elser JJ, Sterner RW, Urabe J (2013) Ecological stoichiometry: An elementary approach using basic principles. Limnol Oceanogr 58: 2219–2236.

[ref117] Hietanen S, Lukkari K (2007) Effects of short-term anoxia on benthic denitrification, nutrient fluxes and phosphorus forms in coastal Baltic sediment. Aquat Microb Ecol 49: 293–302.

[ref118] Hjerne O, Hajdu S, Larsson U, Downing A, Winder M (2019) Climate driven changes in timing, composition and size of the Baltic Sea phytoplankton spring bloom. Front Mar Sci 6: 1–15.

[ref119] Hoppe HG (2003) Phosphatase activity in the sea. Hydrobiologia 493: 187–200.

[ref120] Howarth RW, Marino R (2006) Nitrogen as the limiting nutrient for eutrophication in coastal marine ecosystems: evolving views over three decades. Limnol Oceanogr 51: 364–376.

[ref121] Hudson JJ, Taylor WD, Schindler DW (2000) Phosphate concentrations in lakes. Nature 406: 54–56. 10894537 10.1038/35017531

[ref326] Hugerth LW, Larsson J, Alneberg J, Lindh MV, Legrand C, Pinhassi J, Andersson AF (2015) Metagenome-assembled genomes uncover a global brackish microbiome. Genome Biol 16: 1–18. 26667648 10.1186/s13059-015-0834-7 PMC4699468

[ref122] Huisman J, Weissing FJ (1999) Biodiversity of plankton by species oscillations and chaos. Nature 402: 407–410.

[ref123] Humborg C, Ittekkot V, Cociasu A, Bodungen BV (1997) Effect of Danube River Dam on Black Sea biogeochemistry and ecosystem structure. Nature 386: 385–388.

[ref124] Ibelings BW, Portielje R, Lammens EHRR, Noordhuis R, Van Den Berg MS, Joosse W, Meijer ML (2007) Resilience of alternative stable states during the recovery of shallow lakes from eutrophication: Lake Veluwe as a case study. Ecosystems 10: 4–16.

[ref125] Isvánovics V, Shafik HM, Présing M, Juhos S (2000) Growth and phosphate uptake kinetics of the cyanobacterium, Cylindrospermopsis raciborskii (Cyanophyceae) in throughflow cultures. Freshw Biol 43: 257–275.

[ref126] Janssen ABG, Hilt S, Kosten S, de JJM, Paerl HW, Van de DB (2021) Shifting states, shifting services: linking regime shifts to changes in ecosystem services of shallow lakes. Freshw Biol 66: 1–12.

[ref127] Janssen EML, McNeill K (2015) Environmental photoinactivation of extracellular phosphatases and the effects of dissolved organic matter. Environ Sci Technol 49: 889–896. 25495644 10.1021/es504211x

[ref128] Jäntti H, Stange F, Leskinen E, Hietanen S (2011) Seasonal variation in nitrification and nitratereduction pathways in coastal sediments in the Gulf of Finland, Baltic Sea. Aquat Microb Ecol 63: 171–181.

[ref129] Jauzein C, Labry C, Youenou A, Quéré J, Delmas D, Collos Y (2010) Growth and phosphorus uptake by the toxic dinoflagellate Alexandrium catenella (dinophyceae) in response to phosphate limitation. J Phycol 46: 926–936.

[ref130] Jennerjahn TC (2012) Biogeochemical response of tropical coastal systems to present and past environmental change. Earth Sci Rev 114: 19–41.

[ref131] Jenny JP, Francus P, Normandeau A, Lapointe F, Perga ME, Ojala A, Schimmelmann A, Zolitschka B (2016) Global spread of hypoxia in freshwater ecosystems during the last three centuries is caused by rising local human pressure. Glob Change Biol 22: 1481–1489. 10.1111/gcb.13193 26666217

[ref132] Jeppesen E, Meerhoff M, Jacobsen BA, Hansen RS, Søndergaard M, Jensen JP, Lauridsen TL, Mazzeo N, Branco CWCC (2007) Restoration of shallow lakes by nutrient control and biomanipulation - The successful strategy varies with lake size and climate. Hydrobiologia 581: 269–285.

[ref133] Jochimsen MC, Kümmerlin R, Straile D (2013) Compensatory dynamics and the stability of phytoplankton biomass during four decades of eutrophication and oligotrophication. Ecol Lett 16: 81–89. 23050937 10.1111/ele.12018

[ref134] Joint I, Henriksen P, Fonnes GA, Bourne D, Thingstad TF, Riemann B (2002) Competition for inorganic nutrients between phytoplankton and bacterioplankton in nutrient manipulated mesocosms. Aquat Microb Ecol 29: 145–159.

[ref135] Kahru M, Elmgren R, Savchuk OP (2016) Changing seasonality of the Baltic Sea. Biogeosciences 13: 1009–1018.

[ref136] Karl D, Michaels A, Bergman B, Capone D, Carpenter E, Letelier R, Lipschultz F, Paerl H, Sigman D, Stal L (2002) Dinitrogen fixation in the world’s oceans. Biogeochemistry 57–58: 47–98.

[ref137] Karl DM, Björkman KM (2015) Dynamics of dissolved organic phosphorus. In Biogeochemistry of Marine Dissolved Organic Matter, Ed 2. Elsevier, London, pp. 233–334.

[ref138] Karlson AML, Duberg J, Motwani NH, Hogfors H, Klawonn I, Ploug H, Barthel Svedén J, Garbaras A, Sundelin B, Hajdu S et al. (2015) Nitrogen fixation by cyanobacteria stimulates production in Baltic food webs. Ambio 44: 413–426. 26022324 10.1007/s13280-015-0660-x PMC4447702

[ref327] Karstens S, Buczko U, Glatzel S (2015) Phosphorus storage and mobilization in coastal Phragmites wetlands: Influence of local-scale hydrodynamics. Estuar Coast Shelf Sci 164: 124–133.

[ref328] Karstens S, Buczko U, Jurasinski G, Peticzka R, Glatzel S (2016) Impact of adjacent land use on coastal wetland sediments. Sci Total Environ 550: 337–348. 26824269 10.1016/j.scitotenv.2016.01.079

[ref139] Kemp AES, Villareal TA (2018) The case of the diatoms and the muddled mandalas: time to recognize diatom adaptations to stratified waters. Prog Oceanogr 167: 138–149.

[ref140] Kemp WM, Boynton WR, Adolf JE, Boesch DF, Boicourt WC, Brush G, Cornwell JC, Fisher TR, Glibert PM, Hagy JD et al. (2005) Eutrophication of Chesapeake Bay: historical trends and ecological interactions. Mar Ecol Prog Ser 303: 1–29.

[ref141] Kettler GC, Martiny AC, Huang K, Zucker J, Coleman ML, Rodrigue S, Chen F, Lapidus A, Ferriera S, Johnson J et al. (2007) Patterns and implications of gene gain and loss in the evolution of Prochlorococcus. PLoS Genet 3: 2515–2528. 10.1371/journal.pgen.0030231 PMC2151091 18159947

[ref142] Key T, Mccarthy A, Campbell D, Six C, Roy S, Finkel Z (2010) Cell size trade-offs govern light exploitation strategies in marine phytoplankton. Environ Microbiol 12: 95–104. 19735282 10.1111/j.1462-2920.2009.02046.x

[ref143] Kirk JTO (1975) A theoretical analysis of the contribution of algal cells to the attenuation of light within natural waters II. Spherical cells. New Phytol 75: 21–36.

[ref144] Kirk JTO (1976) A theoretical analysis of the contribution of algal cells to the attenuation of light within natural waters III. Cylindrical and spheroidal cells. New Phytol 77: 341–358.

[ref145] Klähn S, Steglich C, Hess WR, Hagemann M (2010) Glucosylglycerate: a secondary compatible solute common to marine cyanobacteria from nitrogen-poor environments. Environ Microbiol 12: 83–94. 19735283 10.1111/j.1462-2920.2009.02045.x

[ref146] Klappenbach JA, Dunbar JM, Schmidt TM (2000) rRNA operon copy number reflects ecological strategies of bacteria. Appl Environ Microbiol 66: 1328–1333. 10742207 10.1128/aem.66.4.1328-1333.2000 PMC91988

[ref147] Klotz A, Georg J, Bučinská L, Watanabe S, Reimann V, Januszewski W, Sobotka R, Jendrossek D, Hess WR, Forchhammer K (2016) Awakening of a dormant cyanobacterium from nitrogen chlorosis reveals a genetically determined program. Curr Biol 26: 2862–2872. 27720620 10.1016/j.cub.2016.08.054

[ref148] Korth F, Deutsch B, Liskow I, Voss M (2012) Uptake of dissolved organic nitrogen by size-fractionated plankton along a salinity gradient from the North Sea to the Baltic Sea. Biogeochemistry 111: 347–360.

[ref149] Kraemer BM, Anneville O, Chandra S, Dix M, Kuusisto E, Livingstone DM, Rimmer A, Schladow SG, Silow E, Sitoki LM et al. (2015) Morphometry and average temperature affect lake stratification responses to climate change. Geophys Res Lett 42: 4981–4988.

[ref150] Kremp A, Oja J, Letortorec AH, Hakanen P, Tahvanainen P, Tuimala J, Suikkanen S (2016) Diverse seed banks favour adaptation of microalgal populations to future climate conditions. Environ Microbiol 18: 679–691. 26913820 10.1111/1462-2920.13070

[ref151] Krom MD, Thingstad TF, Brenner S, Carbo P, Drakopoulos P, Fileman TW, GAF F, Groom S, Herut B, Kitidis V et al. (2005) Summary and overview of the CYCLOPS P addition Lagrangian experiment in the Eastern Mediterranean. Deep Sea Res Part II Top Stud Oceanogr 52: 3090–3108.

[ref152] Kufel L, Kufel I (2002) Chara beds acting as nutrient sinks in shallow lakes—a review. Aquat Bot 72: 249–260.

[ref153] Laamanen MJ, Forsström L, Sivonen K (2002) Diversity of Aphanizomenon flos-aquae (cyanobacterium) populations along a Baltic Sea salinity gradient. Appl Environ Microbiol 68: 5296–5303. 12406717 10.1128/AEM.68.11.5296-5303.2002 PMC129895

[ref154] Labry C, Delmas D, Herbland A (2005) Phytoplankton and bacterial alkaline phosphatase activities in relation to phosphate and DOP availability within the Gironde plume waters (Bay of Biscay). J Exp Mar Biol Ecol 318: 213–225.

[ref155] Lacour T, Larivière J, Babin M (2017) Growth, Chl a content, photosynthesis, and elemental composition in polar and temperate microalgae. Limnol Oceanogr 62: 43–58.

[ref156] Lampert W, Sommer U (2007) Limnoecology, Ed 2nd. Oxford University Press, Oxford.

[ref157] Lampert W, Sommer U (2013) Limnoecology, Ed 2nd. Oxford University Press, Oxford.

[ref158] Larsson J, Celepli N, Ininbergs K, Dupont CL, Yooseph S, Bergman B, Ekman M (2014) Picocyanobacteria containing a novel pigment gene cluster dominate the brackish water Baltic Sea. ISME J 8: 1892–1903. 24621524 10.1038/ismej.2014.35 PMC4139726

[ref159] Lavaud J, Six C, Campbell DA (2016) Photosystem II repair in marine diatoms with contrasting photophysiologies. Photosynth Res 127: 189–199. 26156125 10.1007/s11120-015-0172-3

[ref160] Lee GF (1973) Role of phosphorus in eutrophication and diffuse source control. Water Res 7: 111–128.

[ref161] Lee ZM, Steger L, Corman JR, Neveu M, Poret-Peterson AT, Souza V, Elser JJ (2015) Response of a stoichiometrically imbalanced ecosystem to manipulation of nutrient supplies and ratios. PLoS One 10: e0123949. 25881015 10.1371/journal.pone.0123949 PMC4399942

[ref162] Lee ZMP, Bussema C, Schmidt TM (2009) rrn DB: documenting the number of rRNA and tRNA genes in bacteria and archaea. Nucleic Acids Res 37: 489–493. 10.1093/nar/gkn689 PMC2686494 18948294

[ref163] Lewis WM, Wurtsbaugh WA, Paerl HW (2011) Rationale for control of anthropogenic nitrogen and phosphorus to reduce eutrophication of inland waters. Environ Sci Technol 45: 10300–10305. 22070635 10.1021/es202401p

[ref164] Lewitus AJ, Kana TM (1995) Light respiration in six estuarine phytoplankton species: contrasts under photoautotrophic and mixotrophic growth conditions. J Phycol 31: 754–761.

[ref165] Li F, Wu Y, Hutchins DA, Fu F, Gao K (2016) Physiological responses of coastal and oceanic diatoms to diurnal fluctuations in seawater carbonate chemistry under two CO_2_ concentrations. Biogeosciences 13: 6247–6259.

[ref166] Li G, Brown CM, Jeans JA, Donaher NA, Mccarthy A, Campbell DA (2015) The nitrogen costs of photosynthesis in a diatom under current and future pCO2. New Phytol 205: 533–543. 25256155 10.1111/nph.13037

[ref167] Li J, Plouchart D, Zastepa A, Dittrich M (2019a) Picoplankton accumulate and recycle polyphosphate to support high primary productivity in coastal Lake Ontario. Sci Rep 9: 1–10. 31862973 10.1038/s41598-019-56042-5 PMC6925121

[ref168] Li M, Peng C, Zhou X, Yang Y, Guo Y, Shi G, Zhu Q (2019b) Modeling global riverine DOC flux dynamics from 1951 to 2015. J Adv Model Earth Syst 11: 514–530.

[ref169] Liang Y, Koester JA, Liefer JD, Irwin AJ, Finkel ZV (2019) Molecular mechanisms of temperature acclimation and adaptation in marine diatoms. ISME J 13: 2415–2425. 31127177 10.1038/s41396-019-0441-9 PMC6776047

[ref170] Liang Z, Soranno PA, Wagner T (2020) The role of phosphorus and nitrogen on chlorophyll a: evidence from hundreds of lakes. Water Res 185: 116236. 32739700 10.1016/j.watres.2020.116236

[ref171] Liénart C, Garbaras A, Qvarfordt S, Sysoev AÖ, Höglander H, Walve J, Schagerström E, Eklöf J, Karlson AML (2021) Long-term changes in trophic ecology of blue mussels in a rapidly changing ecosystem. Limnol Oceanogr 66: 694–710.

[ref172] Lin S, Litaker RW, Sunda WG (2016) Phosphorus physiological ecology and molecular mechanisms in marine phytoplankton. J Phycol 52: 10–36. 26987085 10.1111/jpy.12365

[ref173] Litchman E, Nguyen BLVV (2008) Alkaline phosphatase activity as a function of internal phosphorus concentration in freshwater phytoplankton. J Phycol 44: 1379–1383. 27039852 10.1111/j.1529-8817.2008.00598.x

[ref174] Liu H, Chen M, Zhu F, Harrison PJ (2016) Effect of diatom silica content on copepod grazing, growth and reproduction. Front Mar Sci 3: 1–7.

[ref175] Liu Y, Villalba G, Ayres RU, Schroder H (2008) Global phosphorus flows and environmental impacts from a consumption perspective. J Ind Ecol 12: 229–247.

[ref176] Loebl M, Cockshutt AM, Campbell DA, Finkel ZV (2010) Physiological basis for high resistance to photoinhibition under nitrogen depletion in *Emiliania huxleyi*. Limnol Oceanogr 55: 2150–2160.

[ref177] Lürling M (2003) Phenotypic plasticity in the green algae Desmodesmus and Scenedesmus with special reference to the induction of defensive morphology. Ann Limnol Int J Limnol 39: 85–101.

[ref178] Lürling M (2020) Grazing resistance in phytoplankton. Hydrobiologia 3: 237–249.

[ref179] Malerba ME, Heimann K, Connolly SR (2016) Nutrient utilization traits vary systematically with intraspecific cell size plasticity. Funct Ecol 30: 1745–1755.

[ref180] Mallin MA, McIver MR, Ensign SH, Cahoon LB (2004) Photosynthetic and heterotrophic impacts of nutrient loading to blackwater streams. Ecol Appl 14: 823–838.

[ref181] Mallin MA, Paerl HW, Rudek J, Bates PW (1993) Regulation of estuarine primary production by watershed rainfall and river flow. Mar Ecol Prog Ser 93: 199–203.

[ref182] Marañón E, Cermeño P, López-Sandoval DC, Rodríguez-Ramos T, Sobrino C, Huete-Ortega M, Blanco JM, Rodríguez J (2013) Unimodal size scaling of phytoplankton growth and the size dependence of nutrient uptake and use. Ecol Lett 16: 371–379. 23279624 10.1111/ele.12052

[ref183] Marañón E, Lorenzo MP, Cermeño P, Mouriño-Carballido B (2018) Nutrient limitation suppresses the temperature dependence of phytoplankton metabolic rates. ISME J 12: 1836–1845. 29695860 10.1038/s41396-018-0105-1 PMC6018665

[ref184] Martin P, Dyhrman ST, Lomas MW, Poulton NJ, Van BAS (2014) Accumulation and enhanced cycling of polyphosphate by Sargasso Sea plankton in response to low phosphorus. Proc Natl Acad Sci 111: 8089–8094. 24753593 10.1073/pnas.1321719111 PMC4050623

[ref185] Martin-Jézéquel V, Hildebrand M, Brzezinski MA (2000) Silicon metabolism in diatoms: implications for growth. J Phycol 36: 821–840.

[ref186] Martiny AC, Huang Y, Li W (2009a) Occurrence of phosphate acquisition genes in Prochlorococcus cells from different ocean regions. Environ Microbiol 11: 1340–1347. 19187282 10.1111/j.1462-2920.2009.01860.x

[ref187] Martiny AC, Kathuria S, Berube PM (2009b) Widespread metabolic potential for nitrite and nitrate assimilation among Prochlorococcus ecotypes. Proc Natl Acad Sci U S A 106: 10787–10792. 19549842 10.1073/pnas.0902532106 PMC2705535

[ref188] McCarthy MJ, Gardner WS, Lehmann MF, Bird DF (2013) Implications of water column ammonium uptake and regeneration for the nitrogen budget in temperate, eutrophic Missisquoi Bay, Lake Champlain (Canada/USA). Hydrobiologia 718: 173–188.

[ref189] McCormick PV, Shuford RBE, Chimney MJ (2006) Periphyton as a potential phosphorus sink in the Everglades Nutrient Removal Project. Ecol Eng 27: 279–289.

[ref190] McQuatters-Gollop A, Raitsos DE, Edwards M, Pradhan Y, Mee LD, Lavender SJ, Attrill MJ (2007) A long-term chlorophyll dataset reveals regime shift in North Sea phytoplankton biomass unconnected to nutrient levels. Limnol Oceanogr 52: 635–648.

[ref191] Molinos-Senante M, Hernández-Sancho F, Sala-Garrido R, Garrido-Baserba M (2011) Economic feasibility study for phosphorus recovery processes. Ambio 40: 408–416. 21809783 10.1007/s13280-010-0101-9 PMC3357736

[ref192] Molot LA, Watson SB, Creed IF, Trick CG, Mccabe SK, Verschoor MJ, Sorichetti RJ, Powe C, Venkiteswaran JJ, Schiff SL (2014) A novel model for cyanobacteria bloom formation: the critical role of anoxia and ferrous iron. Freshw Biol 59: 1323–1340.

[ref193] Moore LR, Ostrowski M, Scanlan DJ, Feren K, Sweetsir T (2005) Ecotypic variation in phosphorus-acquisition mechanisms within marine picocyanobacteria. Aquat Microb Ecol 39: 257–269.

[ref194] Motwani NH, Gorokhova E (2013) Mesozooplankton grazing on picocyanobacteria in the Baltic Sea as inferred from molecular diet analysis. PLoS One 8: e79230. 24260175 10.1371/journal.pone.0079230 PMC3832457

[ref195] Mulholland MR, Lomas MW (2008) Nitrogen uptake and assimilation. In Nitrogen in the Marine Environment. Elsevier, London, pp 303–384.

[ref196] Muro-Pastor MI, Reyes JC, Florencio FJ (2001) Cyanobacteria perceive nitrogen status by sensing intracellular 2-oxoglutarate levels. J Biol Chem 276: 38320–38328. 11479309 10.1074/jbc.M105297200

[ref197] Murphy CD, Roodvoets MS, Austen EJ, Dolan A, Barnett A, Campbell DA (2017) Photoinactivation of Photosystem II in Prochlorococcus and Synechococcus. PLoS One 12: e0168991. 28129341 10.1371/journal.pone.0168991 PMC5271679

[ref198] Narwani A, Alexandrou MA, Herrin J, Vouaux A, Zhou C, Oakley TH, Cardinale BJ (2015) Common ancestry is a poor predictor of competitive traits in freshwater green algae. PLoS One 10: e0137085. 26348482 10.1371/journal.pone.0137085 PMC4562640

[ref199] Naumann E (1931) Limnologische Terminologie. Urban & Schwarzenberg, Berlin.

[ref200] Nausch M (1998) Alkaline phosphatase activities and the relationship to inorganic phosphate in the Pomeranian Bight (southern Baltic Sea). Aquat Microb Ecol 16: 87–94. 10.1007/s002489900112 9852505

[ref201] Nausch M, Nausch G, Mohrholz V, Siegel H, Wasmund N (2012) Is growth of filamentous cyanobacteria supported by phosphate uptake below the thermocline? Estuar Coast Shelf Sci 99: 50–60.

[ref202] Newell SE, Davis TW, Johengen TH, Gossiaux D, Burtner A, Palladino D, McCarthy MJ (2019) Reduced forms of nitrogen are a driver of non-nitrogen-fixing harmful cyanobacterial blooms and toxicity in Lake Erie. Harmful Algae 81: 86–93. 30638502 10.1016/j.hal.2018.11.003

[ref203] Ni G, Zimbalatti G, Murphy CD, Barnett AB, Arsenault CM, Li G, Cockshutt AM, Campbell DA (2017) Arctic Micromonas uses protein pools and non-photochemical quenching to cope with temperature restrictions on Photosystem II protein turnover. Photosynth Res 131: 203–220. 27639727 10.1007/s11120-016-0310-6 PMC5247552

[ref204] Nieminen M, Piirainen S, Sikström U, Löfgren S, Marttila H, Sarkkola S, Laurén A, Finér L (2018) Ditch network maintenance in peat-dominated boreal forests: Review and analysis of water quality management options. Ambio 47: 535–545. 29589199 10.1007/s13280-018-1047-6 PMC6072635

[ref205] Nishida I, Murata N (1996) Chilling sensitivity in plants and cyanobacteria: the crucial contribution of membrane lipids. Annu Rev Plant Physiol Plant Mol Biol 47: 541–568. 15012300 10.1146/annurev.arplant.47.1.541

[ref206] Nürnberg GK (2009) Assessing internal phosphorus load: problems to be solved. Lake Reserv Manag 25: 419–432.

[ref207] Olofsson M, Robertson EK, Edler L, Arneborg L, Whitehouse MJ, Ploug H (2019) Nitrate and ammonium fluxes to diatoms and dinoflagellates at a single cell level in mixed field communities in the sea. Sci Rep 9: 1–12. 30723237 10.1038/s41598-018-38059-4 PMC6363804

[ref208] O’Neil JM, Davis TW, Burford MA, Gobler CJ (2012) The rise of harmful cyanobacteria blooms: the potential roles of eutrophication and climate change. Harmful Algae 14: 313–334.

[ref209] Orsini L, Vanoverbeke J, Swillen I, Mergeay J, De L (2013) Drivers of population genetic differentiation in the wild: isolation by dispersal limitation, isolation by adaptation and isolation by colonization. Mol Ecol 22: 5983–5999. 24128305 10.1111/mec.12561

[ref210] Ostermaier V, Schanz F, Köster O, Kurmayer R (2012) Stability of toxin gene proportion in red-pigmented populations of the cyanobacterium Planktothrix during 29 years of re-oligotrophication of Lake Zürich. BMC Biol 10: 100. 23216925 10.1186/1741-7007-10-100 PMC3534634

[ref211] Ostrowski M, Mazard S, Tetu SG, Phillippy K, Johnson A, Palenik B, Paulsen IT, Scanlan DJ (2010) PtrA is required for coordinate regulation of gene expression during phosphate stress in a marine Synechococcus. ISME J 4: 908–921. 20376102 10.1038/ismej.2010.24

[ref212] Paerl HW (2006) Assessing and managing nutrient-enhanced eutrophication in estuarine and coastal waters: interactive effects of human and climatic perturbations. Ecol Eng 26: 40–54.

[ref213] Paerl HW, Hall NS, Calandrino ES (2011) Controlling harmful cyanobacterial blooms in a world experiencing anthropogenic and climatic-induced change. Sci Total Environ 409: 1739–1745. 21345482 10.1016/j.scitotenv.2011.02.001

[ref214] Parmesan C (2006) Ecological and evolutionary responses to recent climate change. Annu Rev Ecol Evol Syst 37: 637–669.

[ref215] Parslow JS, Harrison PJ, Thompson PA (1984) Saturated uptake kinetics: transient response of the marine diatom *Thalassiosira pseudonana* to ammonium, nitrate, silicate or phosphate starvation. Mar Biol 83: 51–59.

[ref216] Pasciak W, Gavis J (1974) Transport limitation of nutrient uptake in phytoplankton. Limnol Oceanogr 19: 881–888.

[ref217] Pastuszak M, Nagel K, Grelowski A (2003) Nutrient dynamics in the Pomeranian Bay (southern Baltic): impact of the Oder River outflow. Estuaries 26: 1238–1254.

[ref218] Peter KH, Sommer U (2013) Phytoplankton cell size reduction in response to warming mediated by nutrient limitation. PLoS One 8: 1–6. 10.1371/journal.pone.0071528 PMC3764198 24039717

[ref219] Peter KH, Sommer U (2015) Interactive effect of warming, nitrogen and phosphorus limitation on phytoplankton cell size. Ecol Evol 5: 1011–1024. 25798219 10.1002/ece3.1241 PMC4364816

[ref220] Ploug H (2008) Cyanobacterial surface blooms formed by Aphanizomenon sp. and Nodularia spumigena in the Baltic Sea: Small-scale fluxes, pH, and oxygen microenvironments. Limnol Oceanogr 53: 914–921.

[ref221] Ploug H, Stolte W, Jørgensen BB (1999) Diffusive boundary layers of the colony-forming plankton alga, Phaeocystis sp.—implications for nutrient uptake and cellular growth. Limnol Oceanogr 44: 1959–1967.

[ref222] Posch T, Köster O, Salcher MM, Pernthaler J (2012) Harmful filamentous cyanobacteria favoured by reduced water turnover with lake warming. Nat Clim Change 2: 809–813.

[ref223] Post AF, Rihtman B, Wang Q (2012) Decoupling of ammonium regulation and ntcA transcription in the diazotrophic marine cyanobacterium Trichodesmium sp. IMS101. ISME J 6: 629–637. 21938021 10.1038/ismej.2011.121 PMC3280139

[ref224] Price GD, Badger MR, Woodger FJ, Long BM (2008) Advances in understanding the cyanobacterial CO2-concentrating-mechanism (CCM): functional components, Ci transporters, diversity, genetic regulation and prospects for engineering into plants. J Exp Bot 59: 1441–1461. 10.1093/jxb/erm112 17578868

[ref225] Raven JA (2003) Inorganic carbon concentrating mechanisms in relation to the biology of algae. Photosynth Res 77: 155–171. 16228373 10.1023/A:1025877902752

[ref226] Raven JA (2011) The cost of photoinhibition. Physiol Plant 142: 87–104. 21382037 10.1111/j.1399-3054.2011.01465.x

[ref227] Raven JA, Beardall J, Giordano M (2014) Energy costs of carbon dioxide concentrating mechanisms in aquatic organisms. Photosynth Res 121: 111–124. 24390639 10.1007/s11120-013-9962-7

[ref228] Redfield AC, Ketchum BH, Richards FA (1963) The influence of organisms on the composition of sea water. In The Sea: Ideas and Observations on Progress in the Study of the Seas. Wiley Interscience, New York, pp. 26–77.

[ref229] Reusch TBH, Boyd PW (2013) Experimental evolution meets marine phytoplankton. Evolution 67: 1849–1859. 23815643 10.1111/evo.12035

[ref230] Reusch TBH, Dierking J, Andersson HC, Bonsdorff E, Carstensen J, Casini M, Czajkowski M, Hasler B, Hinsby K, Hyytiäinen K et al. (2018) The Baltic Sea as a time machine for the future coastal ocean. Sci Adv 4: eaar8195. 29750199 10.1126/sciadv.aar8195 PMC5942908

[ref231] Reynolds CS (2012) Environmental requirements and habitat preferences of phytoplankton: chance and certainty in species selection. Bot Mar 55: 1–17.

[ref232] Rhee G-Y (1974) Phosphate uptake under nitrate limitation by Scenedesmus sp. and its ecological implications. J Phycol 10: 470–475.

[ref233] Riegman R (1995) Nutrient-related selection mechanisms in marine phytoplankton communities and the impact of eutrophication on the planktonic food web. Water Sci Technol 32: 63–75.

[ref234] Riemann B, Carstensen J, Dahl K, Fossing H, Hansen JW, Jakobsen HH, Josefson AB, Krause-Jensen D, Markager S, Stæhr PA et al. (2016) Recovery of Danish coastal ecosystems after reductions in nutrient loading: a holistic ecosystem approach. Estuaries Coasts 39: 82–97.

[ref235] Ritchie RJ, Trautman DA, Larkum AWD (2001) Phosphate limited cultures of the cyanobacterium Synechococcus are capable of very rapid, opportunistic uptake of phosphate. New Phytol 152: 189–201.

[ref236] Roberts S, Shelly K, Beardall J (2008) Interactions among phosphate uptake, photosynthesis, and chlorophyll fluorescence in nutrient-limited cultures of the chlorophyte microalga Dunaliella tertiolecta. J Phycol 44: 662–669. 27041424 10.1111/j.1529-8817.2008.00515.x

[ref237] Rocap G, Larimer FW, Lamerdin J, Malfatti S, Chain P, Ahlgren NA, Arellano A, Coleman M, Hauser L, Hess WR et al. (2003) Genome divergence in two Prochlorococcus ecotypes reflects oceanic niche differentiation. Nature 424: 1042–1047. 12917642 10.1038/nature01947

[ref238] Rogers MW, Allen MS (2012) An ecosystem model for exploring lake restoration effects on fish communities and fisheries in Florida. Restor Ecol 20: 612–622.

[ref239] Rohwer F, Thurber RV (2009) Viruses manipulate the marine environment. Nature 459: 207–212. 19444207 10.1038/nature08060

[ref240] Royal Society (Great Britain) (2005) Ocean Acidification Due to Increasing Atmospheric Carbon Dioxide. Royal Society, London.

[ref241] Rykaczewski RR, Dunne JP (2010) Enhanced nutrient supply to the California Current Ecosystem with global warming and increased stratification in an earth system model. Geophys Res Lett 37: 1–5.

[ref242] Rynearson TA, Armbrust EV (2004) Genetic differentiation among populations of the planktonic marine diatom Ditylum brightwellii (Bacillariophyceae). J Phycol 40: 34–43.

[ref243] Sas H (1989) Lake Restoration by Reduction of Nutrient Loading. Expectations, Experiences, Extrapolations, Ed 1st. Academia Verlag Richarz, St Augustin.

[ref244] Scheffer M, Rinaldi S, Mur LR (1997) On the dominance of filamentous blue-green algae in shallow lakes. Ecology 78: 272–282.

[ref245] Schiewer U (2007) Darß-Zingst Boddens, Northern Rügener Boddens and Schlei. In U Schiewer, MM Caldwell, G Heldmaier, RB Jackson, OL Lange, HA Mooney, E-D Schulze, U Sommer, eds, Ecology of Baltic Coastal Waters, Ed 1st. Springer, Berlin, Heidelberg, pp. 35–86.

[ref246] Schindler DW (2006) Recent advances in the understanding and management of eutrophication. Limnol Oceanogr 51: 356–363.

[ref247] Schindler DW, Carpenter SR, Chapra SC, Hecky RE, Orihel DM (2016) Reducing phosphorus to curb lake eutrophication is a success. Environ Sci Technol 50: 8923–8929. 27494041 10.1021/acs.est.6b02204

[ref248] Schmidt K, Birchill AJ, Atkinson A, Brewin RJW, Clark JR, Hickman AE, Johns DG, Lohan MC, Milne A, Pardo S et al. (2020) Increasing picocyanobacteria success in shelf waters contributes to long-term food web degradation. Glob Change Biol 26: 5574–5587. 10.1111/gcb.15161 32506810

[ref249] Schuergers N, Lenn T, Kampmann R, Meissner MV, Esteves T, Temerinac-Ott M, Korvink JG, Lowe AR, Mullineaux CW, Wilde A (2016) Cyanobacteria use micro-optics to sense light direction. Elife 5: 1–16. 10.7554/eLife.12620 PMC4758948 26858197

[ref250] Schwaderer AS, Yoshiyama K, De Tezanos Pinto P, Swenson NG, Klausmeier CA, Litchman E (2011) Eco-evolutionary differences in light utilization traits and distributions of freshwater phytoplankton. Limnol Oceanogr 56: 589–598.

[ref251] Shade A, Peter H, Allison SD, Baho DL, Berga M, Bürgmann H, Huber DH, Langenheder S, Lennon JT, Martiny JBH et al. (2012) Fundamentals of microbial community resistance and resilience. Front Microbiol 3: 1–19. 23267351 10.3389/fmicb.2012.00417 PMC3525951

[ref252] Shen C, Dupont CL, Hopkinson BM (2017) The diversity of CO2-concentrating mechanisms in marine diatoms as inferred from their genetic content. J Exp Bot 68: 3937–3948. 28510761 10.1093/jxb/erx163 PMC5853954

[ref253] Sherman E, Moore JK, Primeau F, Tanouye D (2016) Temperature influence on phytoplankton community growth rates. Global Biogeochem Cycles 30: 440–559.

[ref254] Six C, Finkel ZV, Irwin AJ, Campbell DA (2007a) Light variability illuminates niche-partitioning among marine picocyanobacteria. PLoS One 2: e1341. 18092006 10.1371/journal.pone.0001341 PMC2129112

[ref255] Six C, Finkel ZV, Rodriguez F, Marie D, Partensky F, Campbell DA (2012) Contrasting photoacclimation costs in ecotypes of the marine eukaryotic picoplankter Ostreococcus. Limnol Oceanogr 53: 255–265.

[ref256] Six C, Thomas J-C, Garczarek L, Ostrowski M, Dufresne A, Blot N, Scanlan DJ, Partensky F (2007b) Diversity and evolution of phycobilisomes in marine Synechococcus spp.: a comparative genomics study. Genome Biol 8: R259. 18062815 10.1186/gb-2007-8-12-r259 PMC2246261

[ref257] Smil V (2000) Phosphorus in the environment: natural flows and human interferences. Annu Rev Energy Environ 25: 53–88.

[ref258] Solovchenko A, Gorelova O, Karpova O, Selyakh I, Semenova L, Chivkunova O, Baulina O, Vinogradova E, Pugacheva T, Scherbakov P et al. (2020) Phosphorus feast and famine in cyanobacteria: is luxury uptake of the nutrient just a consequence of acclimation to its shortage? Cell 9: 1933. 10.3390/cells9091933 PMC7564538 32825634

[ref259] Sommer U, Adrian R, De Senerpont Domis L, Elser JJ, Gaedke U, Ibelings B, Jeppesen E, Lürling M, Molinero JC, Mooij WM et al. (2012) Beyond the Plankton Ecology Group (PEG) model: mechanisms driving plankton succession. Annu Rev Ecol Evol Syst 43: 429–448.

[ref260] Sommer U, Gliwicz M, Lampert W, Duncan A (1986) The PEG-model of seasonal succession of planktonic events in fresh waters. Arch Für Hydrobiol 106: 433–471.

[ref261] Sommer U, Peter KH, Genitsaris S, Moustaka-Gouni M (2017) Do marine phytoplankton follow Bergmann’s rule *sensu lato*? Phytoplankton size and temperature. Biol Rev 92: 1011–1026. 27028628 10.1111/brv.12266

[ref262] Søndergaard M, Jeppesen E, Lauridsen TL, Skov C, Van EH, Roijackers R, Lammens E, Portielje R (2007) Lake restoration: successes, failures and long-term effects. J Appl Ecol 44: 1095–1105.

[ref263] Spears BM, Carvalho L, Perkins R, Paterson DM (2008) Effects of light on sediment nutrient flux and water column nutrient stoichiometry in a shallow lake. Water Res 42: 977–986. 17923145 10.1016/j.watres.2007.09.012

[ref264] Staehr PA, Birkeland MJ (2006) Temperature acclimation of growth, photosynthesis and respiration in two mesophilic phytoplankton species. Phycologia 45: 648–656.

[ref265] Staehr PA, Henriksen P, Markager S (2002) Photoacclimation of four marine phytoplankton species to irradiance and nutrient availability. Mar Ecol Prog Ser 238: 47–59.

[ref266] Stal LJ (2017) The effect of oxygen concentration and temperature on nitrogenase activity in the heterocystous cyanobacterium Fischerella sp. Sci Rep 7: 1–10. 28710405 10.1038/s41598-017-05715-0 PMC5511277

[ref267] Stoddard JL, Larsen DP, Hawkins CP, Johnson RK, Norris RH (2006) Setting expectations for the ecological condition of streams: the concept of reference condition. Ecol Appl 16: 1267–1276. 16937796 10.1890/1051-0761(2006)016[1267:seftec]2.0.co;2

[ref268] Stolte W, Riegman R (1995) Effect of phytoplankton cell size on transient-state nitrate and ammonium uptake kinetics. Microbiology 141: 1221–1229. 33820118 10.1099/13500872-141-5-1221

[ref269] Štrojsová A, Vrba J, Nedoma J, Komárková J, Znachor P (2003) Seasonal study of extracellular phosphatase expression in the phytoplankton of a eutrophic reservoir. Eur J Phycol 38: 295–306.

[ref270] Suzuki Y, Takahashi M (1995) Growth responses of several diatom species isolated from various environments to temperature. J Phycol 31: 880–888.

[ref271] Svensen C, Egge JK, Stiansen JE (2001) Can silicate and turbulence regulate the vertical flux of biogenic matter? A mesocosm study. Mar Ecol Prog Ser 217: 67–80.

[ref272] Swarbrick VJ, Simpson GL, Glibert PM, Leavitt PR (2019) Differential stimulation and suppression of phytoplankton growth by ammonium enrichment in eutrophic hardwater lakes over 16 years. Limnol Oceanogr 64: S130–S149.

[ref273] Syväranta J, Högmander P, Keskinen T, Karjalainen J, Jones RI (2011) Altered energy flow pathways in a lake ecosystem following manipulation of fish community structure. Aquat Sci 73: 79–89.

[ref274] Taipale SJ, Vuorio K, Aalto SL, Peltomaa E, Tiirola M (2019) Eutrophication reduces the nutritional value of phytoplankton in boreal lakes. Environ Res 179: 108836. 31708172 10.1016/j.envres.2019.108836

[ref275] Talmy D, Blackford J, Hardman-Mountford NJ, Polimene L, Follows MJ, Geider RJ (2014) Flexible C:N ratio enhances metabolism of large phytoplankton when resource supply is intermittent. Biogeosciences 11: 4881–4895.

[ref276] Tatters AO, Fu FX, Hutchins DA (2012) High CO 2 and silicate limitation synergistically increase the toxicity of pseudo-nitzschia fraudulenta. PLoS One 7: e32116. 22363805 10.1371/journal.pone.0032116 PMC3283721

[ref277] Tesson SVM, Montresor M, Procaccini G, Kooistra WHCF (2014) Temporal changes in population structure of a marine planktonic diatom. PLoS One 9: 1–23. 10.1371/journal.pone.0114984 PMC4266644 25506926

[ref278] Thamatrakoln K, Hildebrand M (2008) Silicon uptake in diatoms revisited: a model for saturable and nonsaturable uptake kinetics and the role of silicon transporters. Plant Physiol 146: 1397–1407. 18162598 10.1104/pp.107.107094 PMC2259041

[ref279] Thingstad TF, Bellerby RGJ, Bratbak G, Børsheim KY, Egge JK, Heldal M, Larsen A, Neill C, Nejstgaard J, Norland S et al. (2008) Counterintuitive carbon-to-nutrient coupling in an Arctic pelagic ecosystem. Nature 455: 387–390. 18716617 10.1038/nature07235

[ref280] Thomas MK, Kremer CT, Klausmeier CA, Litchman E (2012) A global pattern of thermal adaptation in marine phytoplankton. Science 338: 1085–1088. 23112294 10.1126/science.1224836

[ref281] Thompson PA, Guo M-X, Harrison PJ (1992) Effects of variation in temperature. I. On the biochemical composition of eight species of marine phytoplankton. J Phycol 28: 481–488.

[ref282] Tilman D (1977) Resource competition between plankton algae: an experimental and theoretical approach. Ecology 58: 338–348.

[ref283] Tilman D (1999) Global environmental impacts of agricultural expansion: the need for sustainable and efficient practices. Proc Natl Acad Sci 96: 5995–6000. 10339530 10.1073/pnas.96.11.5995 PMC34218

[ref284] Tilzer MM (1987) Light-dependence of photosynthesis and growth in cyanobacteria: implications for their dominance in eutrophic lakes. N Z J Mar Freshw Res 21: 401–412.

[ref285] Ting C, Rocap G, King J, Chisholm S (2002) Cyanobacterial photosynthesis in the oceans: the origins and significance of divergent light-harvesting strategies. Trends Microbiol 10: 134–142. 11864823 10.1016/s0966-842x(02)02319-3

[ref286] Tipping E, Benham S, Boyle JF, Crow P, Davies J, Fischer U, Guyatt H, Helliwell R, Jackson-Blake L, Lawlor AJ et al. (2014) Atmospheric deposition of phosphorus to land and freshwater. Environ Sci Process Impacts 16: 1608–1617. 24526176 10.1039/c3em00641g

[ref287] Toepel J, Welsh E, Summerfield TC, Pakrasi HB, Sherman LA (2008) Differential transcriptional analysis of the cyanobacterium Cyanothece sp. strain ATCC 51142 during light-dark and continuous-light growth. J Bacteriol 190: 3904–3913. 18390663 10.1128/JB.00206-08 PMC2395039

[ref288] Trombetta T, Vidussi F, Mas S, Parin D, Simier M, Mostajir B (2019) Water temperature drives phytoplankton blooms in coastal waters. PLoS One 14: 1–28. 10.1371/journal.pone.0214933 PMC6450617 30951553

[ref289] Urbach E, Scanlan DJ, Distel DL, Waterbury JB, Chisholm SW (1998) Rapid diversification of marine picophytoplankton with dissimilar light-harvesting structures inferred from sequences of Prochlorococcus and Synechococcus (Cyanobacteria). J Mol Evol 46: 188–201. 9452521 10.1007/pl00006294

[ref290] Ursi S, Pedersén M, Plastino E, Snoeijs P (2003) Intraspecific variation of photosynthesis, respiration and photoprotective carotenoids in Gracilaria birdiae (Gracilariales: Rhodophyta). Mar Biol 142: 997–1007.

[ref291] Vahtera E, Autio R, Kaartokallio H, Laamanen M (2010) Phosphate addition to phosphorus-deficient Baltic Sea plankton communities benefits nitrogen-fixing Cyanobacteria. Aquat Microb Ecol 60: 43–57.

[ref292] van Beusekom JEE, de VN (2012) Dissolved organic phosphorus: An indicator of organic matter turnover? Estuar Coast Shelf Sci 108: 29–36.

[ref293] Van Den Wyngaert S, Möst M, Freimann R, Ibelings BW, Spaak P (2015) Hidden diversity in the freshwater planktonic diatom Asterionella formosa. Mol Ecol 24: 2955–2972. 25919789 10.1111/mec.13218

[ref294] van KC, Lesschen JP, Oenema O (2016) Phosphorus flows and balances of the European Union Member States. Sci Total Environ 542: 1078–1093. 26421756 10.1016/j.scitotenv.2015.08.048

[ref295] van Keulen M, Paling EI, Walker CJ (2003) Effect of planting unit size and sediment stabilization on seagrass transplants in Western Australia. Restor Ecol 11: 50–55.

[ref296] Van Mooy BAS, Rocap G, Fredricks HF, Evans CT, Devol AH (2006) Sulfolipids dramatically decrease phosphorus demand by picocyanobacteria in oligotrophic marine environments. Proc Natl Acad Sci 103: 8607–8612. 16731626 10.1073/pnas.0600540103 PMC1482627

[ref297] Van DP, Bouwman AF, Beusen AHW (2010) Phosphorus demand for the 1970-2100 period: a scenario analysis of resource depletion. Glob Environ Change 20: 428–439.

[ref298] Verhofstad MJJM, Alirangues Núñez MM, Reichman EP, van Donk E, Lamers LPM, Bakker ES (2017) Mass development of monospecific submerged macrophyte vegetation after the restoration of shallow lakes: roles of light, sediment nutrient levels, and propagule density. Aquat Bot 141: 29–38.

[ref299] Vézina S, Vincent WF (1997) Arctic cyanobacteria and limnological properties of their environment: Bylot Island, Northwest Territories, Canada (73°N, 80°W). Polar Biol 17: 523–534.

[ref300] Vincent WF (2000) Cyanobacterial Dominance in the Polar Regions. In The Ecology of Cyanobacteria. Kluwer Academic Publishers, Dordrecht, pp 321–340.

[ref301] Vitousek PM, Aber JD, Howarth RW, Likens GE, Matson PA, Schindler DW, Schlesinger WH, Tilman DG (1997) Human alteration of the global nitrogen cycle: Sources and consequences. Ecol Appl 7: 737–750.

[ref302] Vollenweider RA, Kerekes J (1982) Eutrophication of Waters. Monitoring, Assessment and Control. Organization for Economic Co-Operation and Development (OECD). OECD, Paris, p. 156.

[ref303] Vortmeyer-Kley R, Lünsmann B, Berthold M, Gräwe U, Feudel U (2019) Eddies: fluid dynamical niches or transporters? A case study in the western Baltic Sea. Front Mar Sci 6: 118.

[ref304] Wang H, Mi T, Zhen Y, Jing X, Liu Q, Yu Z (2017) Metacaspases and programmed cell death in Skeletonema marinoi in response to silicate limitation. J Plankton Res 39: 729–743.

[ref305] Wasmund N, Nausch G, Gerth M, Busch S, Burmeister C, Hansen R, Sadkowiak B (2019) Extension of the growing season of phytoplankton in the western Baltic Sea in response to climate change. Mar Ecol Prog Ser 622: 1–16.

[ref306] Wauer G, Gonsiorczyk T, Kretschmer K, Casper P, Koschel R (2005) Sediment treatment with a nitrate-storing compound to reduce phosphorus release. Water Res 39: 494–500. 15644258 10.1016/j.watres.2004.10.017

[ref307] Weisse T, Gröschl B, Bergkemper V (2016) Phytoplankton response to short-term temperature and nutrient changes. Limnologica 59: 78–89.

[ref308] Weyhenmeyer GA, Prairie YT, Tranvik LJ (2014) Browning of boreal freshwaters coupled to carbon-iron interactions along the aquatic continuum. PLoS One 9: e88104. 24505396 10.1371/journal.pone.0088104 PMC3914935

[ref309] White JD, Kaul RRB, Knoll LB, Wilson AE, Sarnellea O (2011) Large variation in vulnerability to grazing within a population of the colonial phytoplankter, *Microcystis aeruginosa*. Limnol Oceanogr 56: 1714–1724.

[ref310] Wilken S, Hoffmann B, Hersch N, Kirchgessner N, Dieluweit S, Rubner W, Hoffmann LJ, Merkel R, Peeken I (2011) Diatom frustules show increased mechanical strength and altered valve morphology under iron limitation. Limnol Oceanogr 56: 1399–1410.

[ref311] Wilken S, Soares M, Urrutia-Cordero P, Ratcovich J, Ekvall MK, Van Donk E, Hansson LA (2018) Primary producers or consumers? Increasing phytoplankton bacterivory along a gradient of lake warming and browning. Limnol Oceanogr 63: S142–S155.

[ref312] Wilson AE, Wilson WA, Hay ME (2006) Intraspecific variation in growth and morphology of the bloom-forming cyanobacterium Microcystis aeruginosa. Appl Environ Microbiol 72: 7386–7389. 16963555 10.1128/AEM.00834-06 PMC1636193

[ref313] Winder M, Cloern JE (2010) The annual cycles of phytoplankton biomass. Philos Trans R Soc B Biol Sci 365: 3215–3226. 10.1098/rstb.2010.0125 PMC2981943 20819814

[ref314] Winter JG, Desellas AM, Fletcher R, Heintsch L, Morley A, Nakamoto L, Utsumi K (2011) Algal blooms in Ontario, Canada: increases in reports since 1994. Lake Reserv Manag 27: 107–114.

[ref315] Wolf KKE, Hoppe CJM, Rost B (2018) Resilience by diversity: large intraspecific differences in climate change responses of an Arctic diatom. Limnol Oceanogr 63: 397–411.

[ref316] Wolf KKE, Romanelli E, Rost B, John U, Collins S, Weigand H, Hoppe CJM (2019) Company matters: the presence of other genotypes alters traits and intraspecific selection in an Arctic diatom under climate change. Glob Change Biol 25: 2869–2884. 10.1111/gcb.14675 PMC6852494 31058393

[ref317] Wynne D, Rhee T-Y (1988) Changes in alkaline phosphatase activity and phosphate uptake in P-limited phytoplankton, induced by light intensity and spectral quality. Hydrobiologia 160: 173–178.

[ref318] Xu X, Jiang B, Tan Y, Costanza R, Yang G (2018) Lake-wetland ecosystem services modeling and valuation: progress, gaps and future directions. Ecosyst Serv 33: 19–28.

[ref319] Yachi S, Loreau M (1999) Biodiversity and ecosystem productivity in a fluctuating environment: the insurance hypothesis. Proc Natl Acad Sci U S A 96: 1463–1468. 9990046 10.1073/pnas.96.4.1463 PMC15485

[ref320] Yoshida T, Hairston NG, Ellner SP (2004) Evolutionary trade-off between defence against grazing and competitive ability in a simple unicellular alga, *Chlorella vulgaris*. Proc R Soc B Biol Sci 271: 1947–1953. 10.1098/rspb.2004.2818 PMC1691804 15347519

[ref321] Young JN, Goldman JAL, Kranz SA, Tortell PD, Morel FMM (2015) Slow carboxylation of Rubisco constrains the maximum rate of carbon fixation during Antarctic phytoplankton blooms. New Phytol 205: 172–181. 25283055 10.1111/nph.13021

[ref322] Zhang TQ, Tan CS, Zheng ZM, Welacky T, Wang YT (2017) Drainage water management combined with cover crop enhances reduction of soil phosphorus loss. Sci Total Environ 586: 362–371. 28189302 10.1016/j.scitotenv.2017.02.025

[ref323] Zhao Y, Quigg A (2014) Nutrient limitation in Northern Gulf of Mexico (NGOM): Phytoplankton communities and photosynthesis respond to nutrient pulse. PLoS One 9: e88732. 24551144 10.1371/journal.pone.0088732 PMC3925166

[ref324] Zhou R, Cao Z, Zhao J (1998) Characterization of HetR protein turnover in Anabaena sp. PCC 7120. Arch Microbiol 169: 417–426. 9560423 10.1007/s002030050592

